# PET Imaging of CXCR4 Receptors in Cancer by a New Optimized Ligand

**DOI:** 10.1002/cmdc.201100320

**Published:** 2011-07-20

**Authors:** Oliver Demmer, Eleni Gourni, Udo Schumacher, Horst Kessler, Hans-Jürgen Wester

**Affiliations:** [a] Institute for Advanced Study and Center of Integrated Protein Science at the Department Chemie, Technische Universität MünchenLichtenbergstraße 4, 85748 Garching (Germany) E-mail: Kessler@tum.de; [b] Lehrstuhl für Pharmazeutische Radiochemie, Technische Universität MünchenWalther-Meißner-Str. 3, 85748 Garching (Germany) E-mail: h.j.wester@tum.de; [c] Institute for Anatomy II: Experimental Morphology, Universitätsklinikum Hamburg-EppendorfMartinistrasse 52, 20246 Hamburg (Germany); [d] Chemistry Department, Faculty of Science, King Abdulaziz UniversityP.O. Box 80203, Jeddah 21589 (Saudi Arabia)

**Keywords:** CXCR4, DOTA, imaging agents, peptides, positron emission tomography, tracers

Nowadays, personalized medicine is considered to be of utmost importance to target the different causes of identical phenotypes.[1–5] For example, cancer of the same type can significantly differ in its biochemical phenotypes and thus its molecular profile between patients. The disease-specific characterization of malignant cells at the molecular level is a prerequisite for targeted therapy and personalized treatment. Positron emission tomography (PET) and its combination with computer tomography (PET/CT) and magnetic resonance tomography (PET/MRT) in modern hybrid systems offer the possibility to localize and quantify biochemical function by means of PET with anatomical (CT) and morphological (MRT) information. For this purpose, radiolabeled probes are used that target, for example, enzyme activities, transport systems, and surface receptors with high affinity and specificity.[6–8] We describe the development of the first gallium-68 (*t*_1/2_=68 min) ligand for the G-protein-coupled receptor CXCR4 and preliminary demonstrate its potential for in vivo imaging of CXCR4 expression using a mouse model with a human small-cell lung cancer xenograft. This ligand offers the possibility to be used as an initial tool for diagnosis in an approach of personalized medicine for treating CXCR4-related cancer.

The chemokine receptor subtype CXCR4 is an attractive target for cancer diagnosis and treatment as it is overexpressed on more than 70 % of human solid tumors, including mammary cancer, prostate cancer, B-cell lymphoma, neuroblastoma, melanoma, cervical adenocarcinoma, and glioma, among others.[9] Moreover, it is involved in three fundamental aspects of cancer: primary tumor growth, cancer cell migration, and establishment of metastatic sites; and therefore, it can be considered an ideal target. Being also a coreceptor for the cellular entry of the HIV, many peptidic and nonpeptidic ligands with different modes of antagonistic activity have been developed.[10–18] These highly CXCR4-specific agents can serve for the introduction of PET-active prosthetic groups. This approach is often complicated by loss of binding affinity, undesired alteration of biodistribution and instability in vivo.[19, 20] A careful optimization of many molecular parameters is necessary to develop a suitable tracer for diagnostic application.

**Figure 1:**
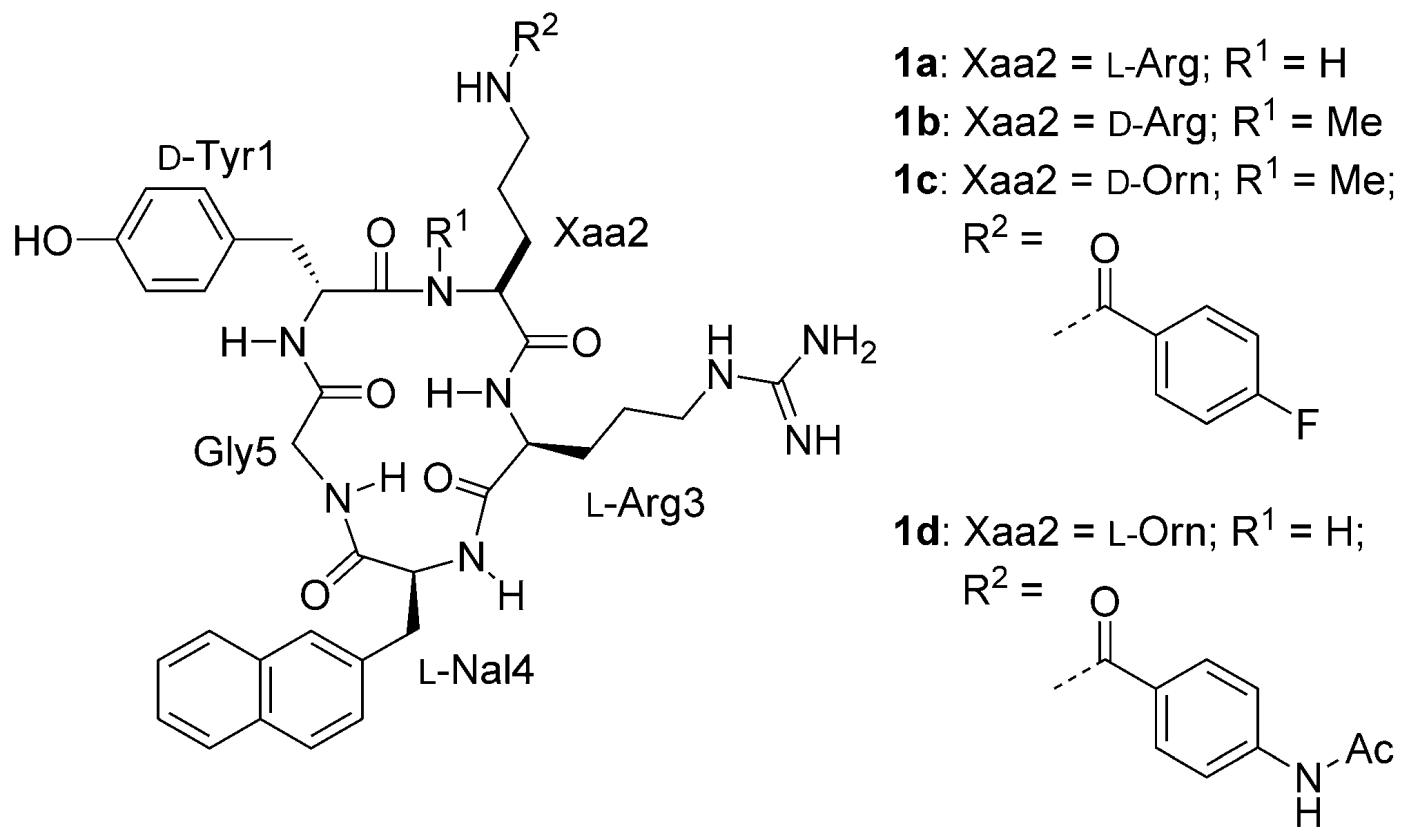
CXCR4 ligands modified to introduce radioisotopes.

As a starting point for the development of the first ^68^Ga-labeled, CXCR4-directed PET probe, we used cyclic pentapeptide **1 a** (Figure 1) developed by Fujii et al. and the later published analogue **1 b**, as it is an inverse agonist of CXCR4.[21–23] Small, cyclic peptides such as these should exhibit high in vivo stability towards enzymatic degradation, especially as they contain d-amino acids and N-methylated peptide bonds.[6] Although allowing first positive imaging experiments, radioiodination of the tyrosine residue increased the lipophilicity and turned out to be unsuccessful for our purpose. Consequently, we investigated the introduction of more hydrophilic groups and focused on the (radio)metal chelator 1,4,7,10-tetraazacyclododecane-1,4,7,10-tetraacetic acid (DOTA) because it can be used in combination with the corresponding radiometals for different imaging techniques like PET (e.g., ^68^Ga^3+^), single photon emission tomography (SPECT; e.g., ^111^In^3+^), or magnetic resonance imaging (MRI; e.g., Gd^3+^, Fe^3+^) and also for radionuclide therapy (e.g., ^177^Lu^3+^, ^90^Y^3+^).

Previous studies of our group and others have shown that all side chains of peptides **1 a** and **1 b** contribute to binding affinity. An attempt to remove the side chain of Arg 3 to introduce anchoring functions in this position resulted in a total loss of activity, whereas substitution of Arg 2 by ornithine (Orn) and its acylated derivatives gave a reduction of only one order of magnitude. Unfortunately, introduction of larger acyl or alkyl substituents on Orn 2 also strongly reduced the affinity (for details see Supporting Information).[24] Unexpectedly, ligands with benzoic acids attached to the Orn 2 side chain retained most of the CXCR4 binding activity for example, **1 d** (Figure 1 and Table 1).[25]

**Table 1:** IC_50_ values for the tested CXCR4 ligands.

Compd	IC_50_[a] [nM]	Compd	IC_50_[a] [nM]
**1 a**	4.3±1.2[b]	**2 a**	150
**1 b**	2±2	**2 b**	44±4
**1 c**	9±1	**2 c**	5±1
**1 d**	22±3		

[a] Values represent the mean±SD of three experiments except where values greater than 100 nM were measured.

[b] Value taken from Reference [15].

**Figure 2:**
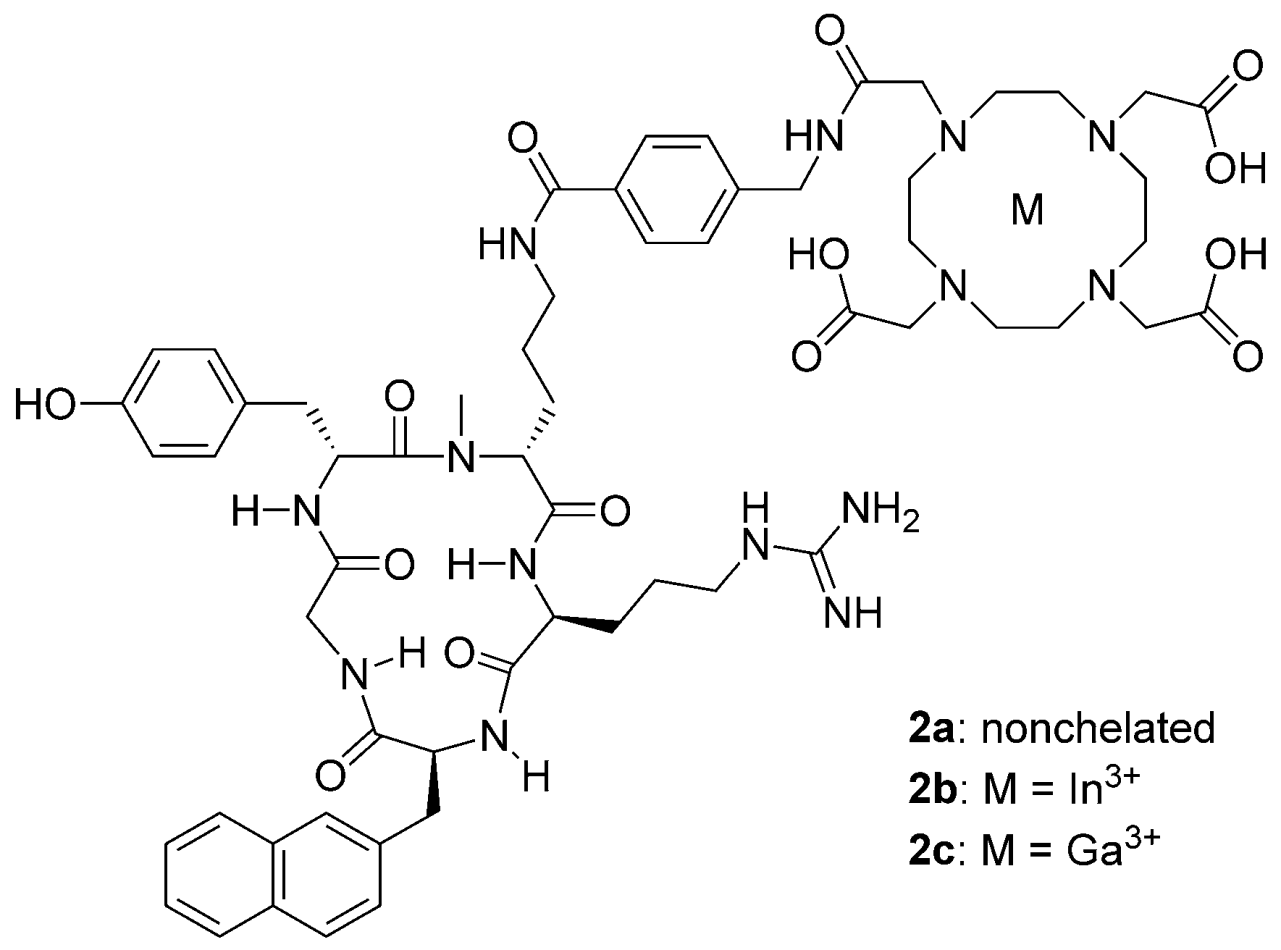
Structures of the best DOTA peptides for imaging. The DOTA moiety is depicted simplified and not representing the actual state of metal ion coordination.

An important affinity improvement was achieved by starting from peptide **1 b**, which differs from **1 a** by an d-arginine residue instead of l-arginine in position 2 and simultaneous N-methylation of the peptide bond between d-Tyr 1-d-Arg 2. We chose DOTA as a complexing moiety as its cyclen scaffold is also found in the CXCR4 drug AMD3100 (Mozobil™), and we hypothesized that we could gain receptor affinity as chelates of AMD3100 have shown to have superior affinity.[26] To attach DOTA, the d-arginine group was again substituted by d-Orn. The type and length of spacer between the peptide and DOTA was optimized in more than 25 compounds (see Supporting Information) to yield the highest affinity compounds **2 a**–**c** (Figure 2 and Table 1). Receptor affinities also depend on the chelation state as well as the type and radii of the metal ion in the complexing moiety.[27–29] Therefore, we tested gallium and indium compounds as they are relevant for imaging purposes and have different ionic radii. While the binding affinities for the free DOTA compound **2 a** and its indium chelate **2 b** are 150 nM and 44±4 nM, respectively, the gallium complex exhibited an affinity of 5±1 nM, which is virtually identical with the unmodified peptides **1 a** and **1 b**.

**Figure 3:**
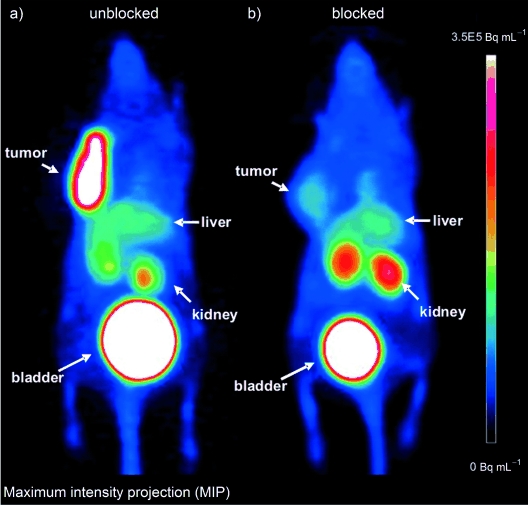
a) PET summation images (90–110 min p.i.) of OH1 h-SCLC tumor-bearing nude mice using [^68^Ga]**2 c** (tracer-only study, injected dose: 230 μCi); b) Competition by co-injection of 50 μg cyclo-(d-Tyr 1-Arg 2-Arg 3-Nal 4-Gly 5) per mouse (injected dose: 300 μCi). The radioactivity scales have been normalized according the injected activity of each mouse.

In vivo testing of [^68^Ga]**2 c** was carried out in nude mice bearing OH-1 human small-cell lung cancer xenografts. The ^68^Ga-labeled ligand accumulated in high levels in CXCR4-expressing tumors and allowed for a high contrasting functional imaging of the CXCR4 receptor status in vivo (Figure 3). Co- injection of 50 μg cyclo-(-d-Tyr 1-Arg 2-Arg 3-Nal 4-Gly 5) or AMD3100 (data not shown) per mouse significantly reduced the tumor uptake, thus demonstrating specificity of CXCR4-mediated tumor binding or [^68^Ga]**2 c.**

Quantitative biodistribution data 60 and 120 min post-injection of [^68^Ga]**2 c** alone or in the presence of 50 μg competitor are summarized in Table 2. High tumor to organ ratios were observed already 1 h post-injection. Furthermore, the results from the classical biodistribution study confirm distribution and specificity of tracer accumulation as observed by the PET imaging study.

**Table 2:** Biodistribution and tumor/muscle ratios of [^68^Ga]**2 c** in OH1 mice at 1 h and 2 h post-injection (p.i.)

Organ	Biodistribution[a] [%ID g^−1^]		Blockade[b]
	1 h p.i. (*n*=8)	2 h p.i. (*n*=3)	1 h p.i. (*n*=4)
Blood	1.08±0.27	0.58±0.14	2.50±0.64
Heart	0.60±0.16	0.29±0.10	1.38±0.52
Lung	1.41±0.26	2.85±2.69	3.25±1.06
Liver	1.85±0.24	1.48±0.31	2.27±0.59
Pancreas	0.30±0.07	0.21±0.02	0.75±0.19
Spleen	0.69±0.09	0.65±0.25	1.03±0.40
Kidney	3.06±0.63	2.07±0.46	7.19±1.83
Adrenal glands	0.83±0.55	0.50±0.15	0.87±0.32
Stomach	0.72±0.26	0.46±0.12	1.40±0.30
Intestine	0.45±0.11	0.36±0.04	1.00±0.32
Muscle	0.38±0.09	0.28±0.17	0.58±0.21
Tumor	6.16±1.16	4.63±1.54	1.88±0.30
Tumor/Muscle	16.55±3.84	18.49±7.29	3.45±1.45

[a] Data are given as the injected dose (%) per gram of tissue and represent the mean±SD of *n*=8, 3 and 4 (see above) experiments.

[b] Determined in the presence of 50 μg cyclo-(d-Tyr 1-Arg 2-Arg 3-Nal 4-Gly 5). All animal experiments were approved by local authorities and are in compliance with the institutions guidelines.

[^68^Ga]**2 c** is the first ^68^Ga-labeled CXCR4 imaging probe and shows excellent in vivo distribution and binding characteristics. The overexpression of CXCR4 in a variety of tumors and its role in organ-specific metastasis recommend the further clinical evaluation of [^68^Ga]**2 c**. This study paves the way for molecular imaging of this important GPCR in animals and man to enable personalized medicine and individualized treatment. Furthermore, chelates of **2 a** with therapeutic nuclides are an obvious choice for possible future endo-radiotherapeutic approaches.
