# Supplementary material for: PET Imaging of CXCR4 Receptors in Cancer by a New Optimized Ligand
Source: ChemMedChem. 2011 Jul 20;6(10):1789–91. doi: 10.1002/cmdc.201100320 (PMC3229844; doi:10.1002/cmdc.201100320)
Supplement: Supplementary file 1 [file cmdc0006-1789-SD1.pdf]

CHEMMEDCHEM
Chemistry & Drug Discovery

# Supporting Information

© Copyright Wiley-VCH Verlag GmbH & Co. KGaA, 69451 Weinheim, 2011

## PET Imaging of CXCR4 Receptors in Cancer by a New Optimized Ligand

Oliver Demmer,[a] Eleni Gourni,[b] Udo Schumacher,[c] Horst Kessler,*[a, d] and Hans-Jürgen Wester*[b]

cmdc_201100320_sm_miscellaneous_information.pdf

# Supporting Information

**Contents:**

Tested compounds and their characterization 2
HPLC-MS spectra of DOTA-labeled compounds 7
Experimental Section 30

**Tested Compounds**

**Figure 1 and Table 1:** Elucidation of an anchoring point starting from FC131 **1a** and initial SAR studies on possible spacers.

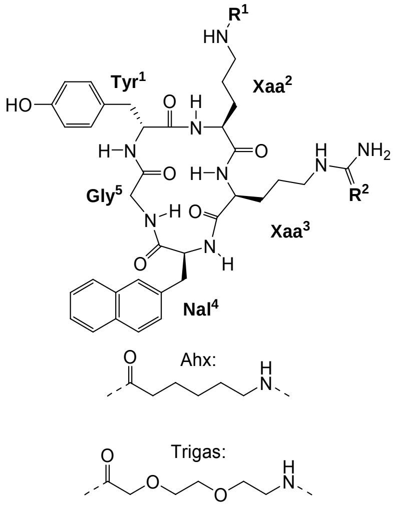


| compd | **R**[1] | **R**[2] | $IC_{50}$ [nM][a] |
|---|---|---|---|
| **1a** | 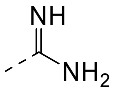 | NH | 4[b] |
| **3** | 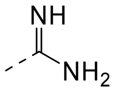 | O | >1000 |
| **4** | 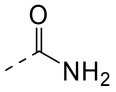 | NH | 35±7 |
| **5** | 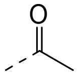 | NH | 29±11 |
| **6** | H | NH | 9±0.1 (19±11[b]) |
| **7** | AhxH | NH | 70±23 |
| **8** | Ahx-AhxH | NH | 947 |
| **9** | Ahx-Ahx-AhxH | NH | 227 |
| **10** | TrigasH | NH | 125 |
| **11** | Trigas-TrigasH | NH | 189 |
| **12** | Trigas-Trigas-TrigasH | NH | 146 |
| **13** | palmitoyl | NH | >1000 |

[a] Mean value of 3 experiments except for values greater than 80 nM; [b] Value from ref. 15

**Figure 2 and Table 2:** In-depth SAR studies of different spacing groups.

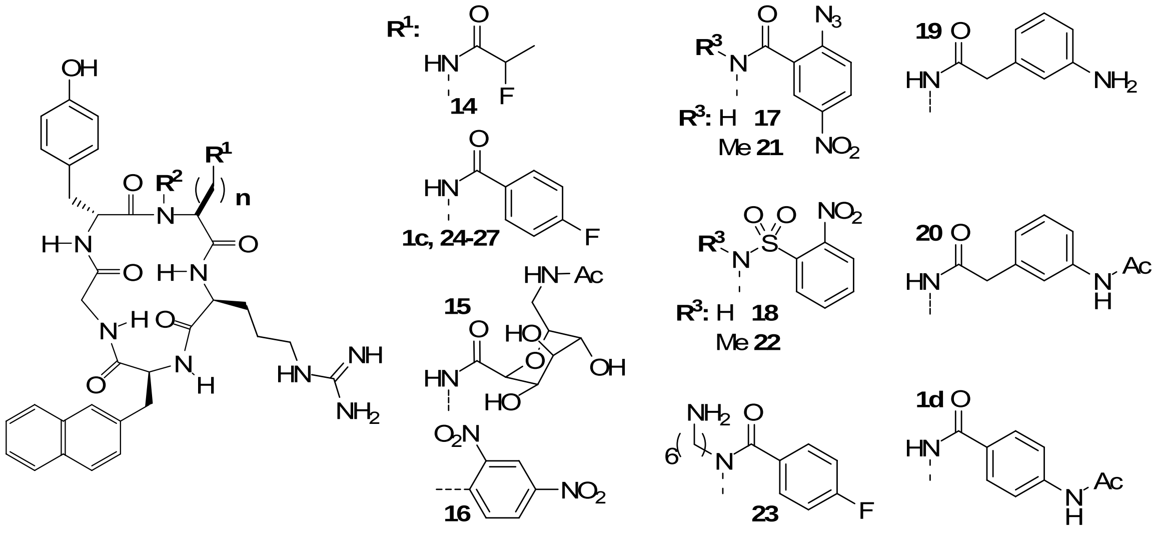


| compd | $R^2$ | n | Orn chirality | $IC_{50}$ [nM][a] |
|---|---|---|---|---|
| **14** | H | 3 | L | 35±15 |
| **1c** | H | 3 | L | 11±2 |
| **15** | H | 3 | L | 360±142 |
| **16** | H | 3 | L | 98±10 |
| **17** | H | 3 | L | 50±9 |
| **18** | H | 3 | L | 130 |
| **19** | H | 3 | L | 61±13 |
| **20** | H | 3 | L | 23±2 |
| **1d** | H | 3 | L | 22±3 |
| **21** | H | 3 | L | 102±8 |
| **22** | H | 3 | L | 520 |
| **23** | H | 3 | L | 124±7 |
| **24** | H | 2 | L | 632 |
| **25** | H | 4 | L | 100±11 |
| **26** | H | 3 | D | 86 |
| **27** | Me | 3 | D | 8.7±0.6 |

[a] Mean value of 3 experiments except for values greater than 80 nM

**Figure 3 and Table 3:** Spacer length and complexation SAR studies of DOTA labeled CXCR4 ligands.

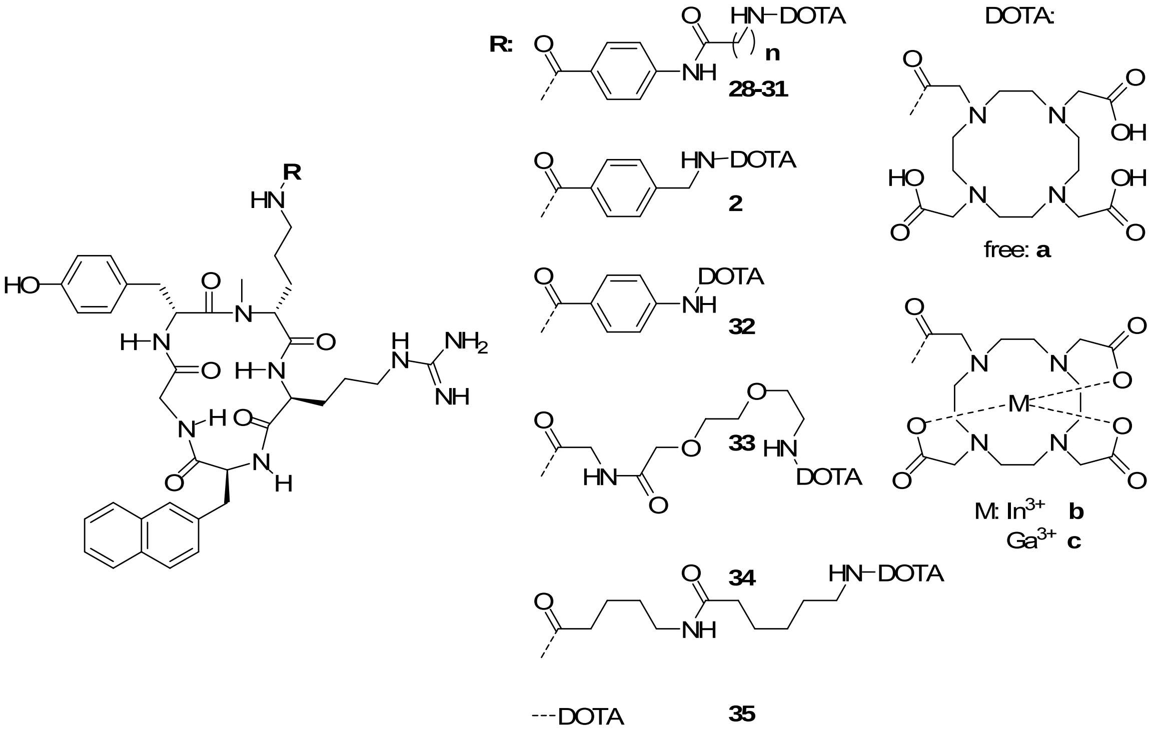


| compd | n | $IC_{50}$ [nM][a] | | |
|---|---|---|---|---|
| | | **a: free DOTA** | **b: $In^{3+}$** | **c: $Ga^{3+}$** |
| **28** | 5 | 1512 | 26.5±22.6 | 30.3±6.5 |
| **29** | 4 | 121±17 | 40.9±21.6 | 14.2±3 |
| **30** | 2 | 41.7±15.5 | 30.4±3.68 | 33.7±3.7 |
| **31** | 1 | 88.3±18.5 | 27.0±7.2 | 16.7±3.3 |
| **2** | - | 150.3 | 44.1±4.2 | 5.0±0.7 |
| **32** | - | 334.7 | 220±162 | 11.5±4.4 |
| **33** | - | 903±439.75 | 457 | - |
| **34** | - | >1000 | 123±25 | 89.7±18.3 |
| **35** | - | 807±477 | >1000 | 289 |

[a] Mean value of 3 experiments except for values greater than 80 nM

## Characterization of tested compounds

**Table 4:** HPLC-MS and $IC_{50}$ values of tested compounds.

| Comp | Exact Mass | HPLC-MS | | $IC_{50}$ [nM] |
|---|---|---|---|---|
| | | $m+H^+$ | $R_t$ [min] | |
| **3** | 730.36 | 731.4 | 7.86 | 1000 |
| **4** | 730.36 | 731.4 | 7.9 | 35±7 |
| **5** | 729.36 | 730.6 | 13.57[a] | 29 ±11 |
| **6** | 687.35 | 688.4 | 7.07 | 9 ±0.1 |
| **7** | 800.43 | 801.7 | 6.66 | 70±23 |
| **8** | 913.52 | 914.7 | 6.82 | 947 |
| **9** | 1026.6 | 1027.8 | 6.96 | 227 |
| **10** | 832.42 | 833.5 | 6.68 | 125 |
| **11** | 977.5 | 978.6 | 6.75 | 189 |
| **12** | 1122.57 | 1123.8 | 6.79 | 146 |
| **13** | 925.58 | 926.9 | 12.39 | >1000 |
| **14** | 761.37 | 762.6 | 13.54[a] | 35 ±13 |
| **1c** | 809.37 | 810.6 | 16.31[a] | 11 ±2 |
| **15** | 918.42 | 919.5 | 7.59 | 594.0 ± 428.5 |
| **16** | 853.35 | 854.4 | 9.49 | 97.7 ± 10.3 |
| **17** | 877.36 | 878.3 | 9.03 | 50.0 ± 8.5 |
| **18** | 872.33 | 873.4 | 9.06 | 125.9 ± 6.2 |
| **19** | 820.4 | 821.7 | 7.08 | 61.4 ± 13.2 |
| **20** | 862.41 | 863.7 | 7.97 | 22.5 ± 1.6 |
| **1d** | 848.4 | 849.6 | 8.72 | 20.2 ± 3.6 |
| **21** | 891.38 | 892.3 | 9.07 | 102.3 ± 7.5 |
| **22** | 886.34 | 887.4 | 9.25 | 520.7 |
| **23** | 908.47 | 909.5 | 7.74 | 124.2 ± 6.6 |
| **24** | 795.35 | 796.6 | 14.82[a] | 632 |
| **25** | 823.38 | 824.4 | 15.38[a] | 99.8 ± 11.0 |
| **26** | 809.37 | 810.4 | 8.45 | 86 |
| **27** | 823.38 | 824.6 | 8.39 | 8.7±0.6 |

[a] Values from the Amersham Pharmacia Biotech Äkta Basic 10F

## DOTA labeled Compounds

**Table 5:** HPLC-MS and $IC_{50}$ values of tested compounds with a DOTA moiety.

| Comp | Exact Mass | HPLC-MS | | $IC_{50}$ [nM] |
|---|---|---|---|---|
| | | $m+H^+$ | $R_t$ [min] | |
| **28a** | 1319.67 | 1320.70 | 7.23 | 1512.3 |
| **28b** | 1431.55 | 1432.6 | 7.58 | 26.5±22.6 |
| **28c** | 1385.57 | 1386.4 | 7.45 | 30.3±6.5 |
| **29a** | 1305.65 | 1306.62 | 7.5 | 121.7±17 |
| **29b** | 1417.53 | 1418.40 | 7.41 | 40.9±21.62 |
| **29c** | 1371.55 | 1372.3 | 7.37 | 14.2±3 |
| **30a** | 1277.62 | 1278.6 | 7.25 | 41.7±15.54 |
| **30b** | 1389.5 | 1390.50 | 7.35 | 30.4±3.68 |
| **30c** | 1343.52 | 1344.40 | 7.27 | 33.66±3.7 |
| **31a** | 1263.6 | 1264.6 | 7.35 | 88.8 ± 18.5 |
| **31b** | 1375.48 | 1376.5 | 7.42 | 27.0±7.2 |
| **31c** | 1329.51 | 1330.4 | 7.21 | 16.7 ± 3.3 |
| **2a** | 1220.6 | 1221.6 | 7.35 | 150.3 |
| **2b** | 1332.48 | 1333.5 | 7.43 | 44.1±4.2 |
| **2c** | 1286.5 | 1287.5 | 7.22 | 5 ±1 |
| **32a** | 1206.58 | 1207.6 | 7.42 | 334.7 |
| **32b** | 1318.46 | 1319.5 | 7.5 | 220±162 |
| **32c** | 1272.48 | 1273.4 | 7.27 | 11.5 ± 4.4 |
| **33a** | 1289.64 | 1290.6 | 6.71 | 903.05±439.75 |
| **33b** | 1401.52 | 1402.6 | 7.19 | 456.8 |
| **34a** | 1285.68 | 1286.6 | 7.34 | >1000 |
| **34b** | 1397.56 | 1398.60 | 7.20 | 123±25 |
| **34c** | 1351.58 | 1352.40 | 7.14 | 89.71±18.3 |
| **35a** | 1087.55 | 1088.6 | 7.26 | 807.5 ± 477.4 |
| **35b** | 1199.43 | 1200.5 | 7.26 | >1000 |
| **35c** | 1153.45 | 1154.5 | 7.14 | 288.9 |

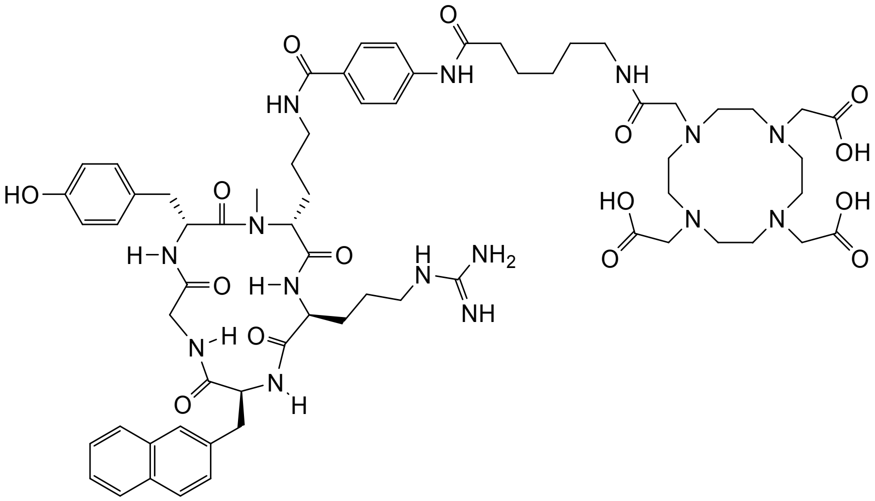

**28a**

**yorn'(Aba, Ahx, DOTA)RNalG**

*cyclo*(-D-Tyr-(α-methyl, δ-4-aminobenzoic acid, 6-aminohexanoic acid, DOTA)-D-Orn-R-Nal-G)

$C_{65}H_{89}N_{15}O_{15}$

Exact Mass: 1319,67

Mol. Wt.: 1320,49

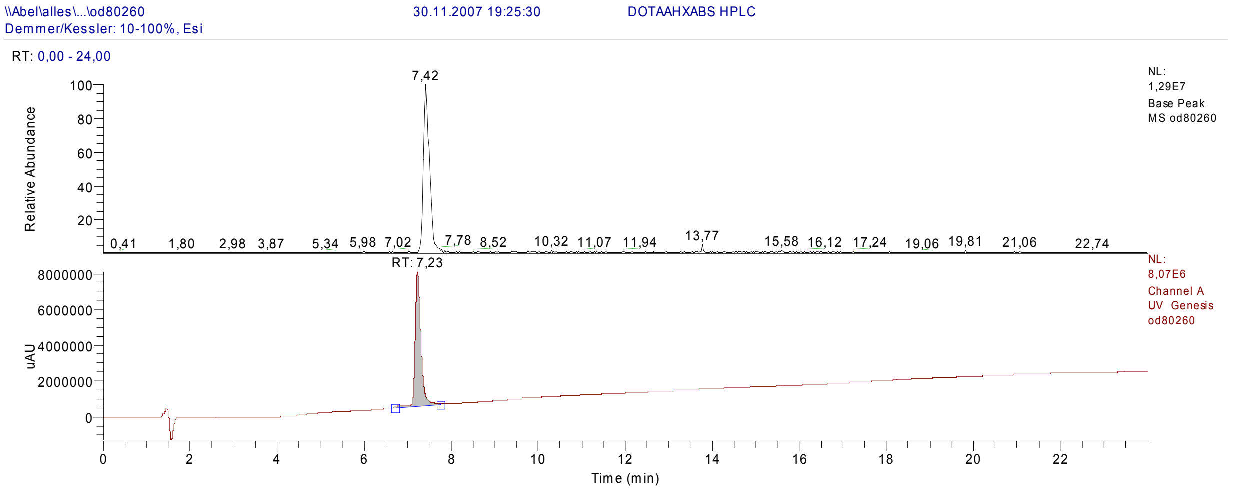


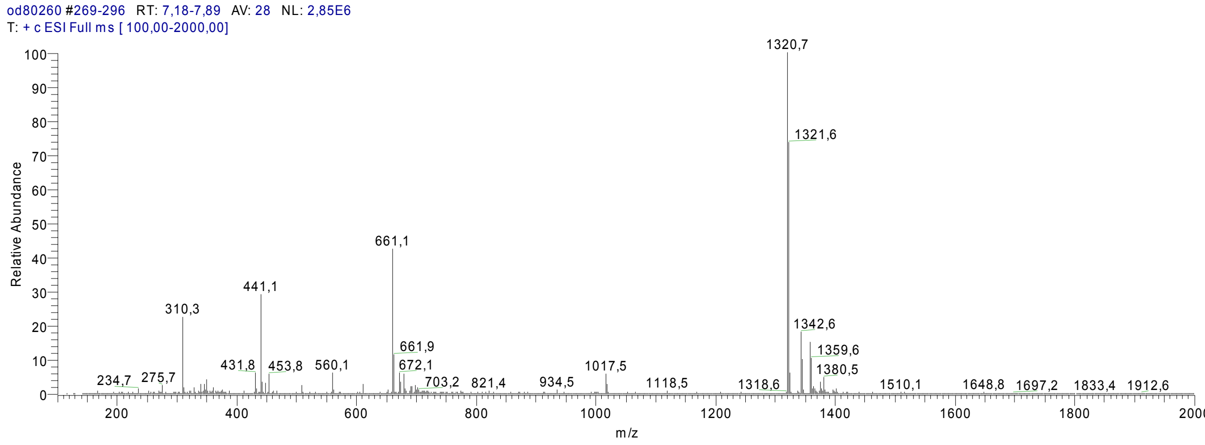

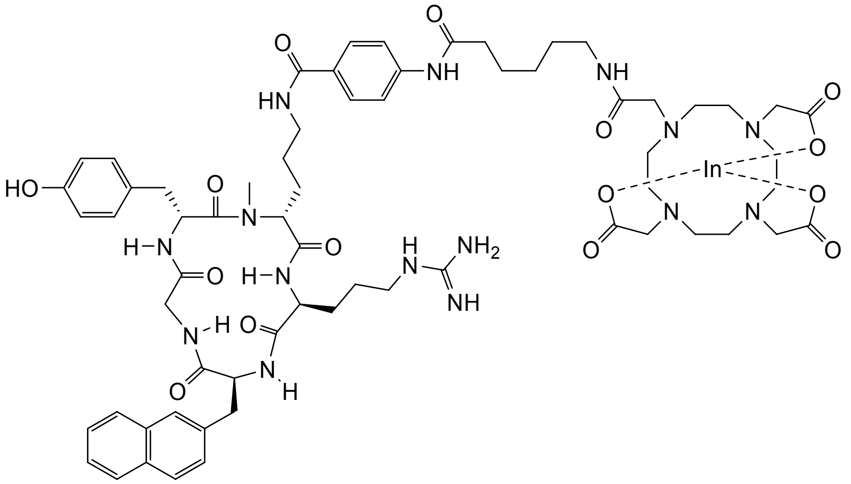

**28b**

**yorn'(Aba, Ahx, DOTA, In)RNalG**

*cyclo*(-D-Tyr-(α-methyl, δ-4-aminobenzoic acid, 6-aminohexanoic acid, DOTA, In)-D-Orn-R-Nal-G)

$C_{65}H_{86}InN_{15}O_{15}$

Exact Mass: 1431,55

Mol. Wt.: 1432,29

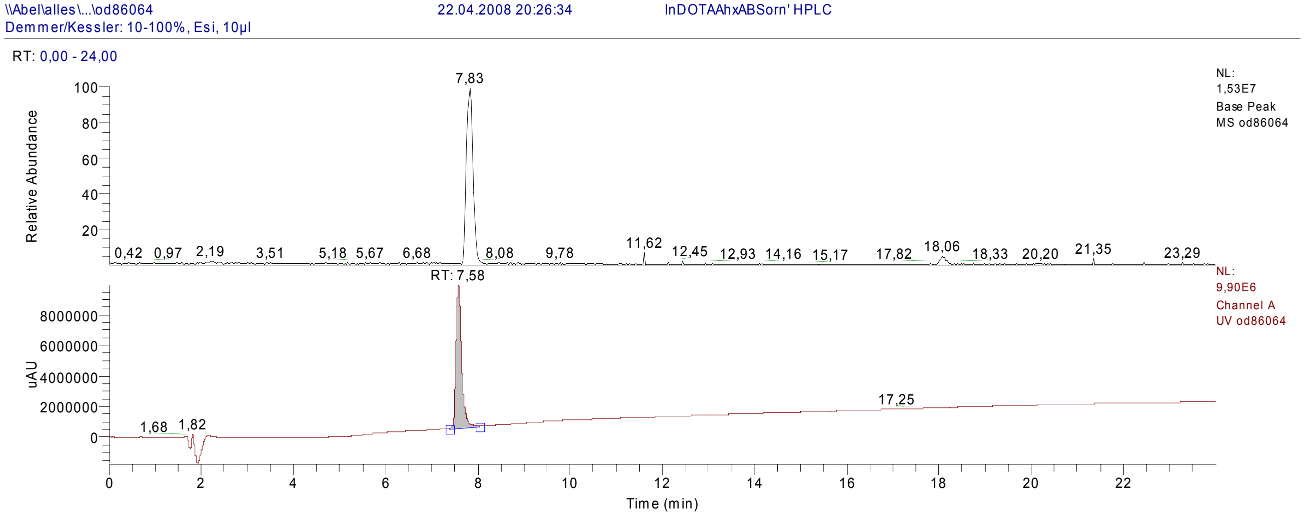


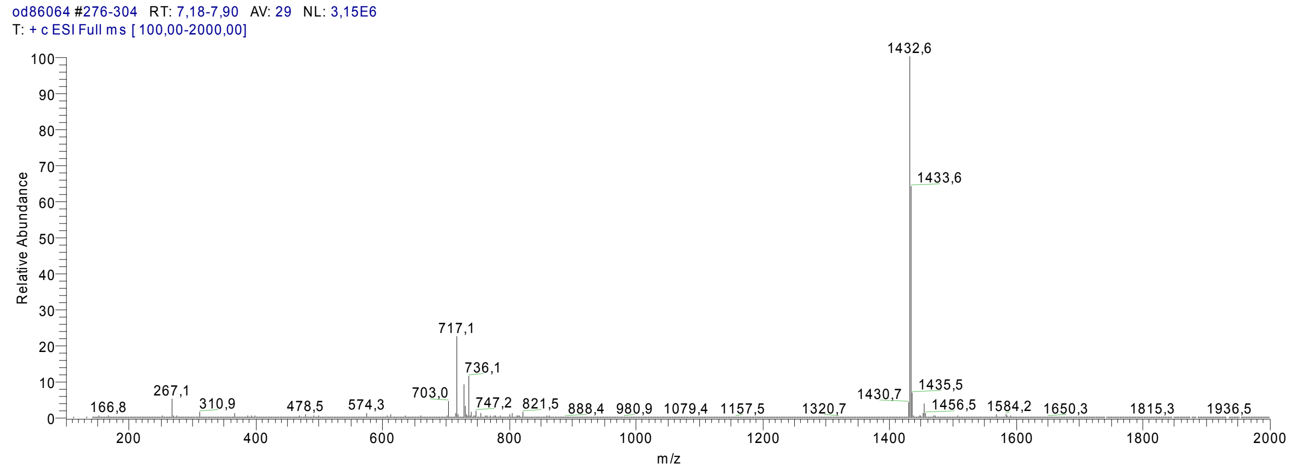

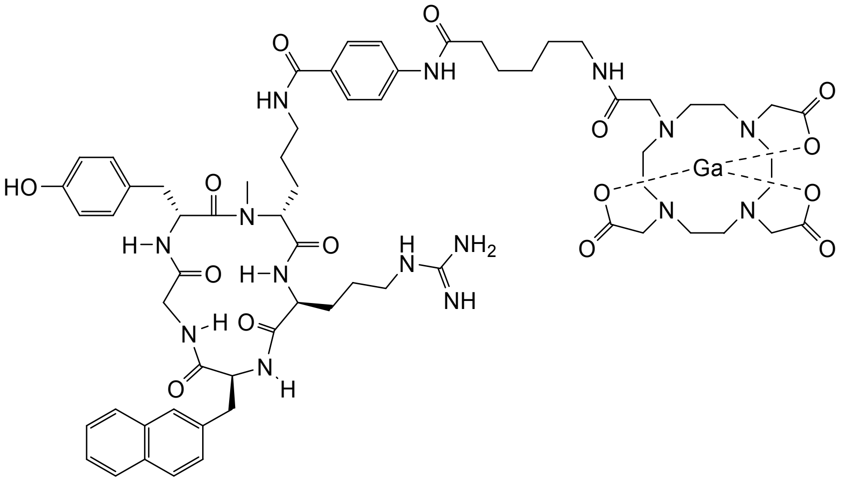


**28c**

**yorn'(Aba, Ahx, DOTA, Ga)RNalG**

*cyclo*(-D-Tyr-(α-methyl, δ-4-aminobenzoic acid, 6-aminohexanoic acid, DOTA, Ga)-D-Orn-R-Nal-G)

$C_{65}H_{86}GaN_{15}O_{15}$

Exact Mass: 1385,57

Mol. Wt.: 1387,19

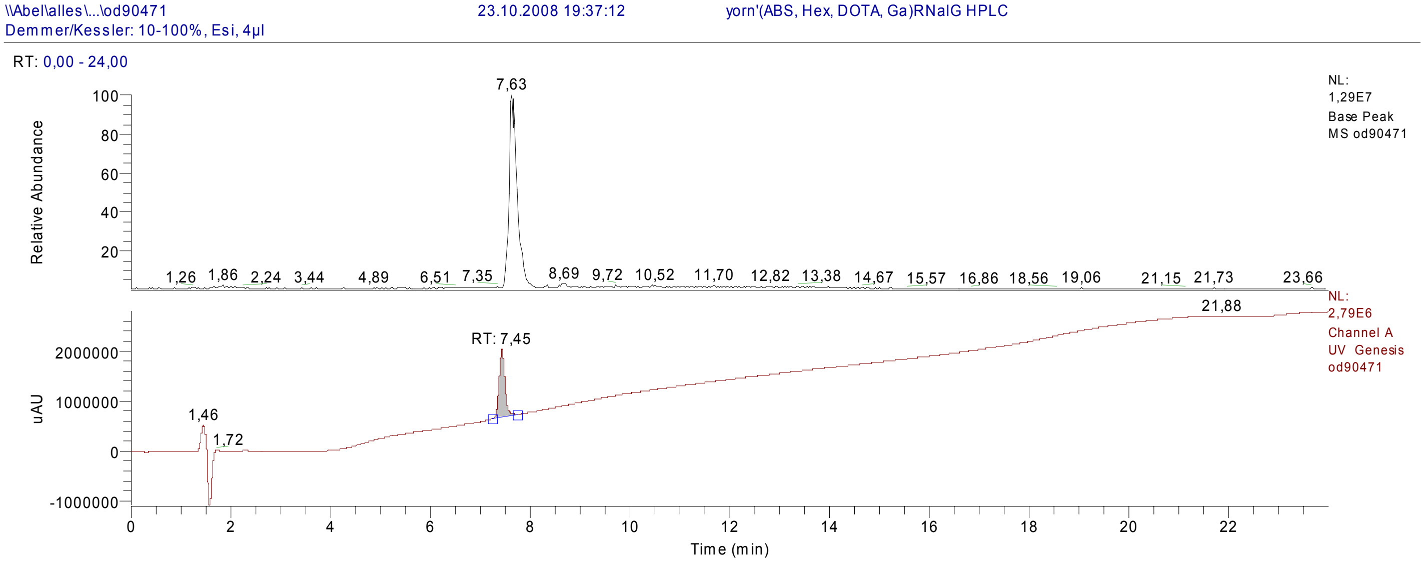


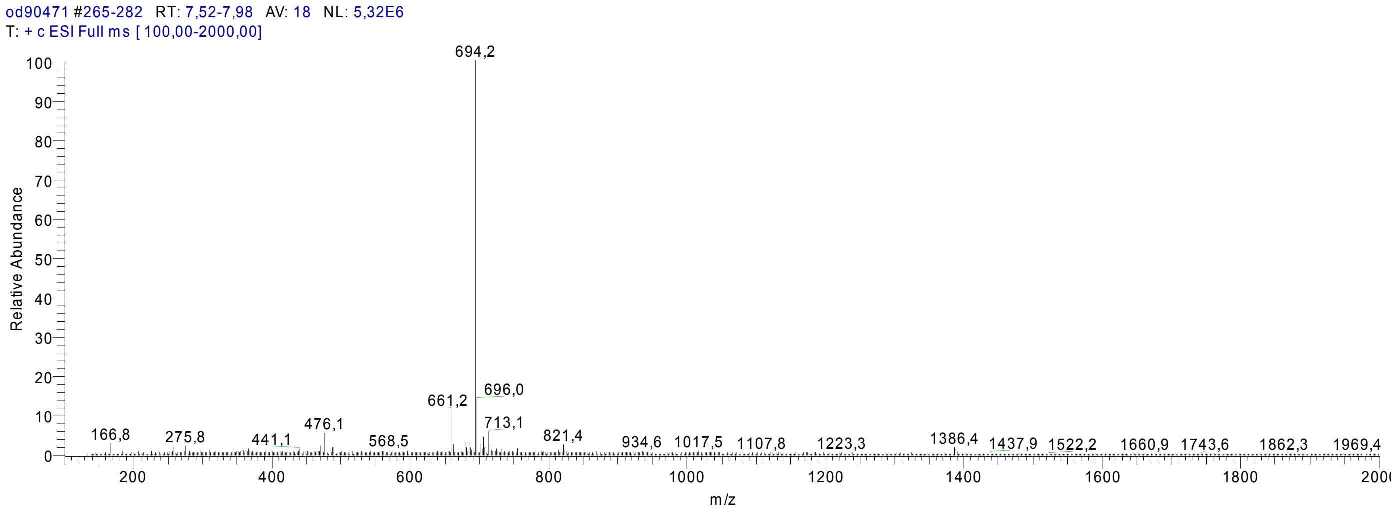

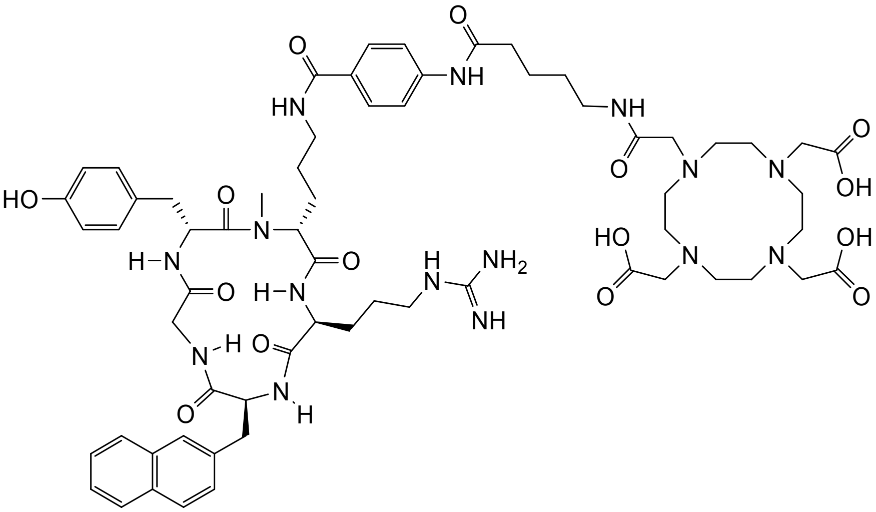

**29a**

**yorn'(Aba, Ava, DOTA)RNalG**

*cyclo*(-D-Tyr-(α-methyl, δ-4-aminobenzoic acid, 5-aminovaleric acid, DOTA)-D-Orn-R-Nal-G)

$C_{64}H_{87}N_{15}O_{15}$

Exact Mass: 1305,65

Mol. Wt.: 1306,47

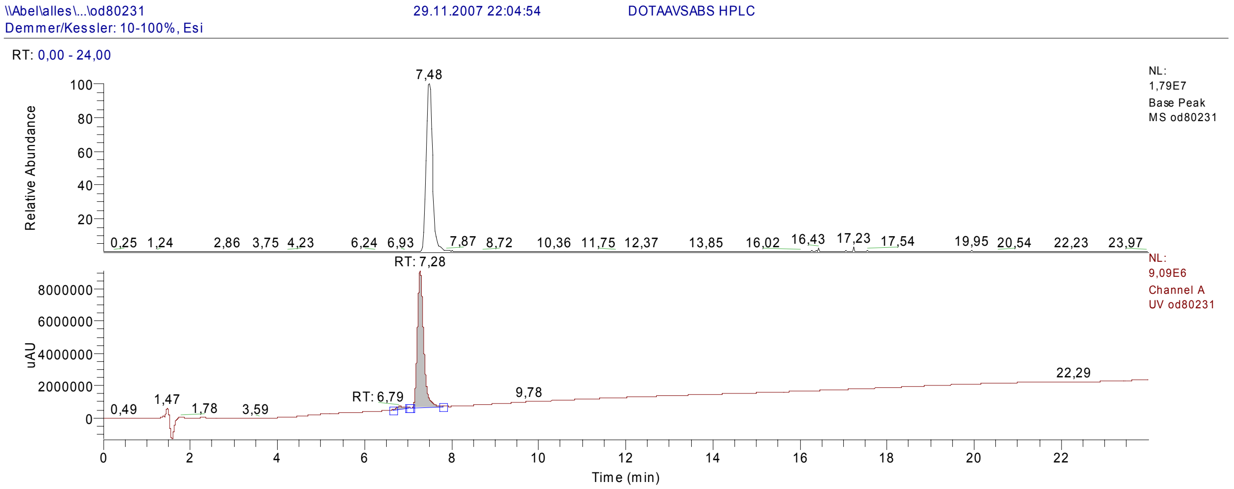


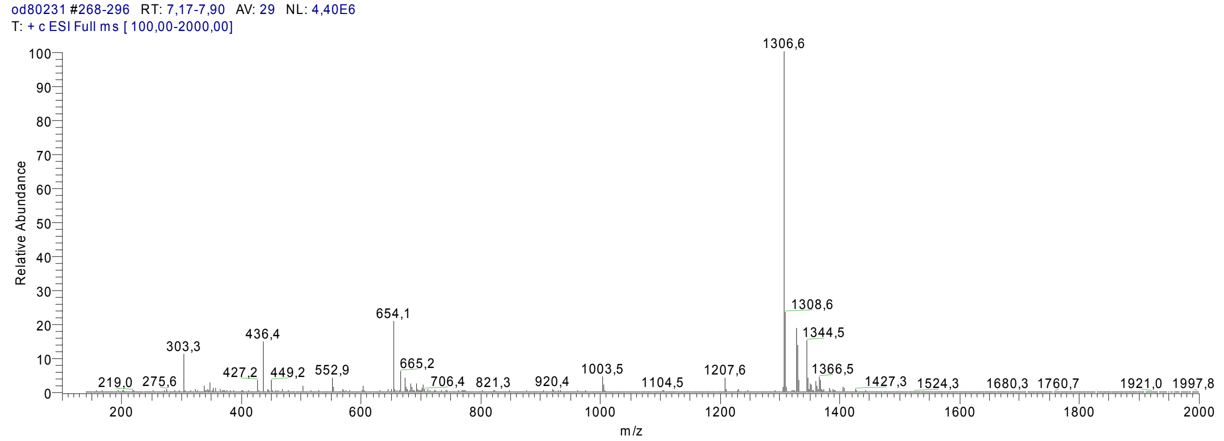

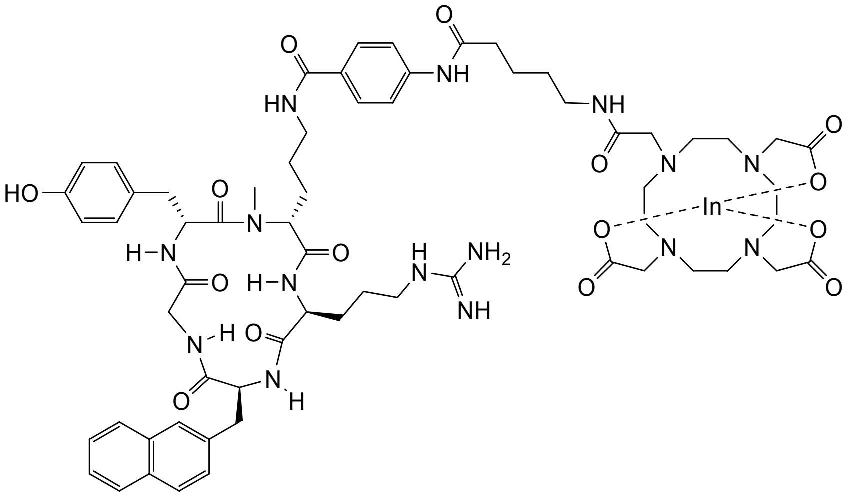

**29b**

**yorn'(Aba, Ava, DOTA, In)RNalG**

*cyclo*(-D-Tyr-(α-methyl, δ-4-aminobenzoic acid, 5-aminovaleric acid, DOTA, In)-D-Orn-R-Nal-G)

$C_{64}H_{84}InN_{15}O_{15}$

Exact Mass: 1417,53

Mol. Wt.: 1418,26

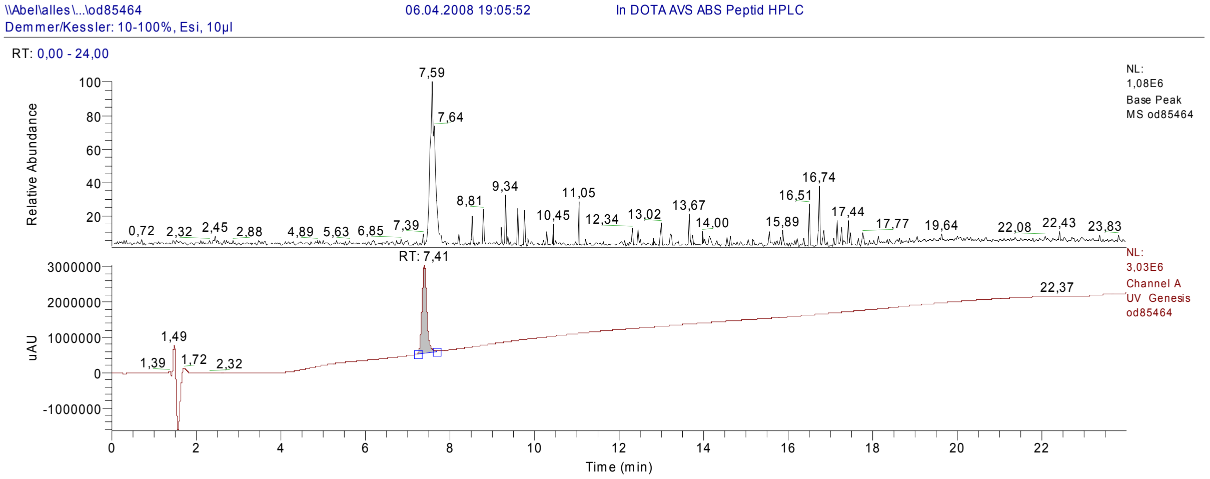


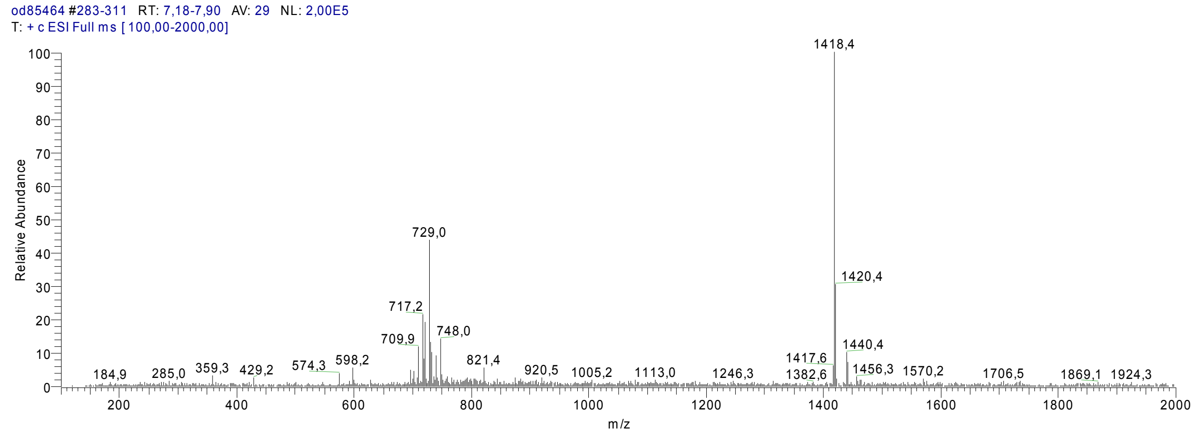

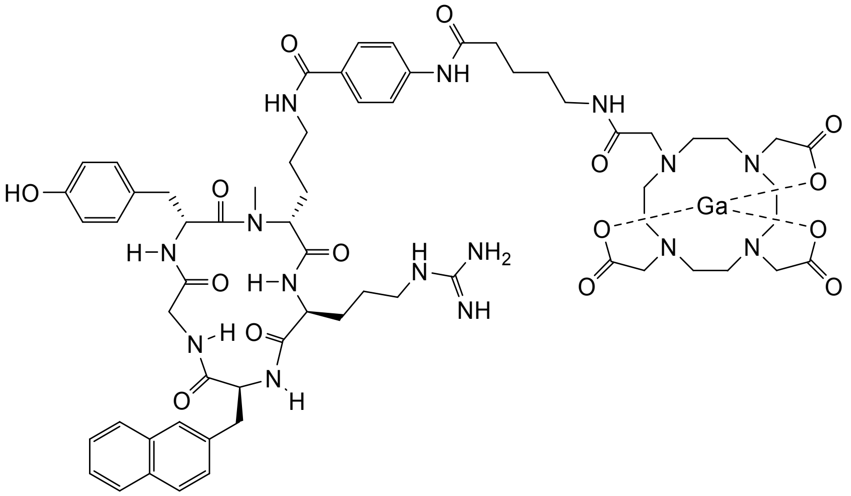

**29c**

**yorn'(Aba, Ava, DOTA, Ga)RNalG**

*cyclo*(-D-Tyr-(α-methyl, δ-4-aminobenzoic acid, 5-aminovaleric acid, DOTA, Ga)-D-Orn-R-Nal-G)

$C_{64}H_{84}GaN_{15}O_{15}$

Exact Mass: 1371,55

Mol. Wt.: 1373,17

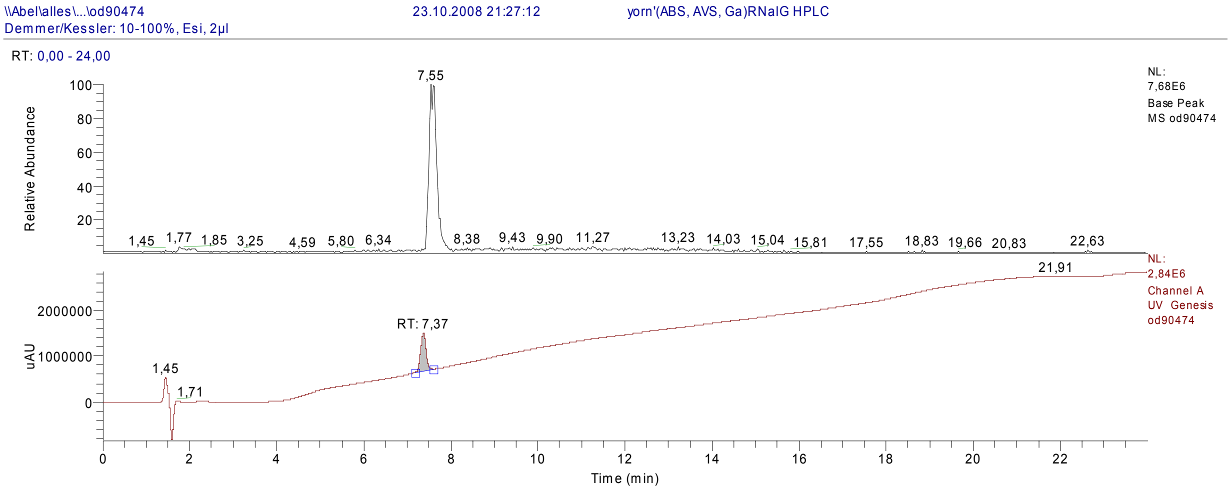


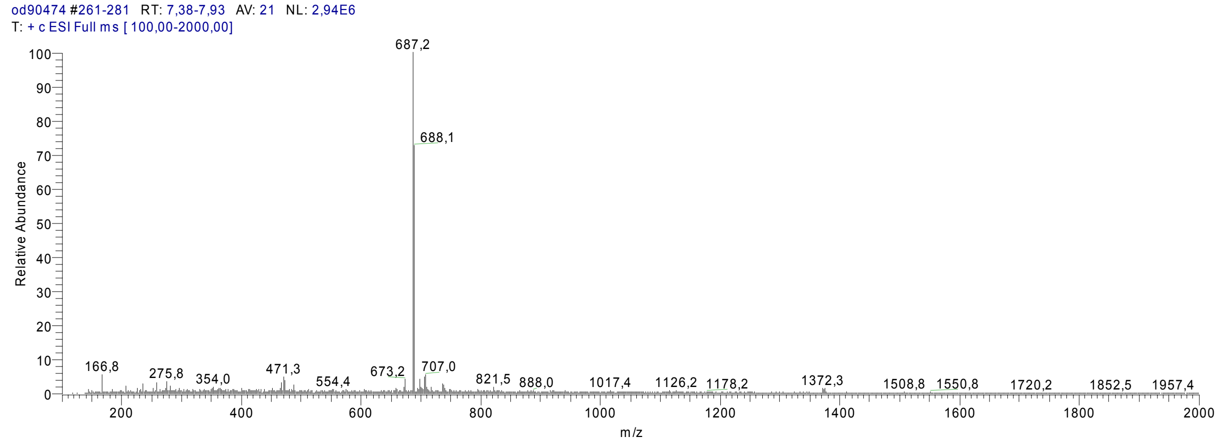

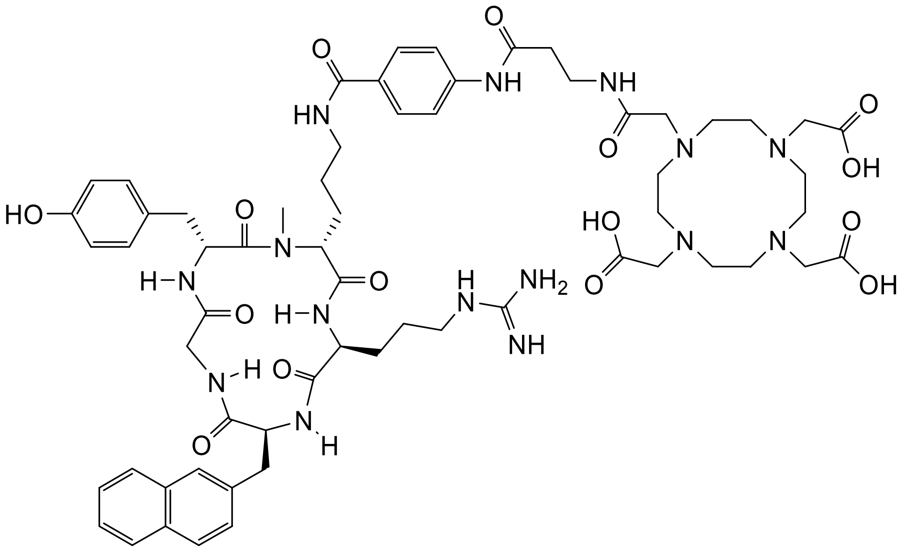

**30a**

**yorn'(Aba, betaAla, DOTA)RNalG**

*cyclo*(-D-Tyr-(α-methyl, δ-4-aminobenzoic acid, beta-alanine, DOTA)-D-Orn-R-Nal-G)

$C_{62}H_{83}N_{15}O_{15}$

Exact Mass: 1277,62

Mol. Wt.: 1278,41

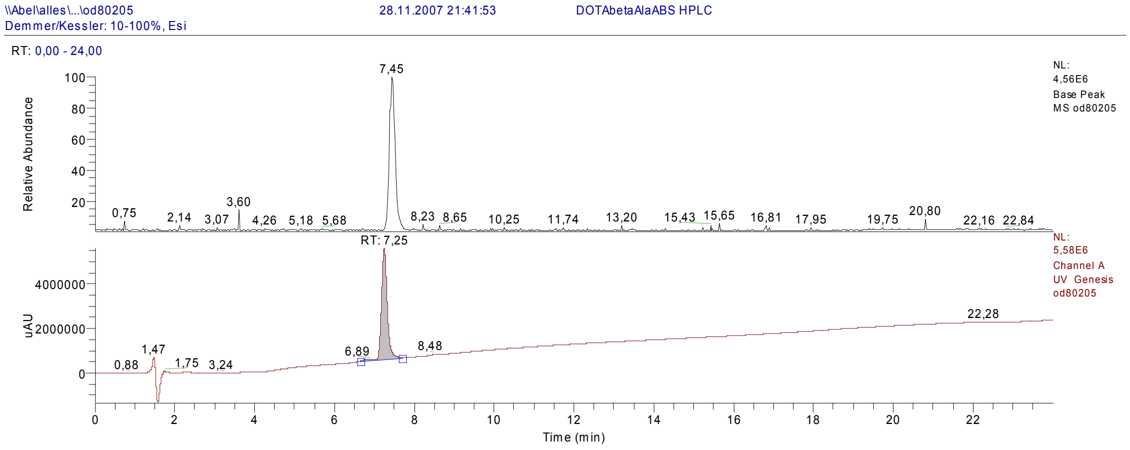


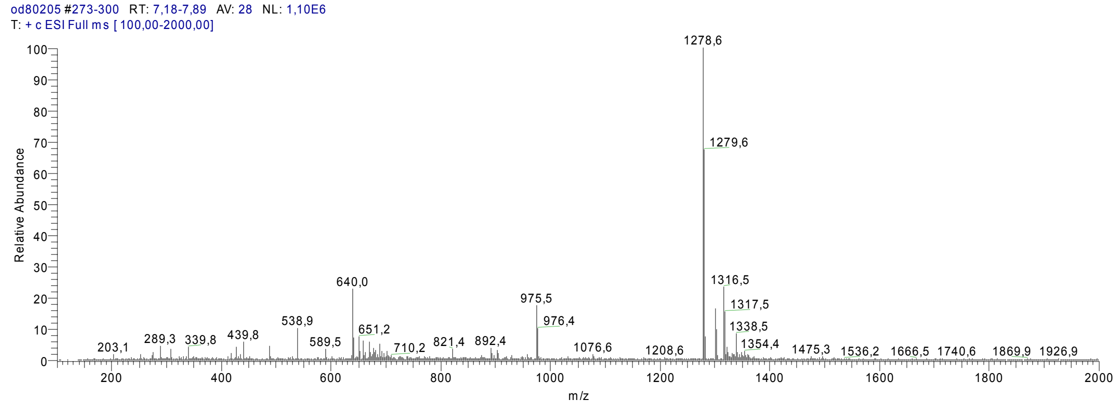

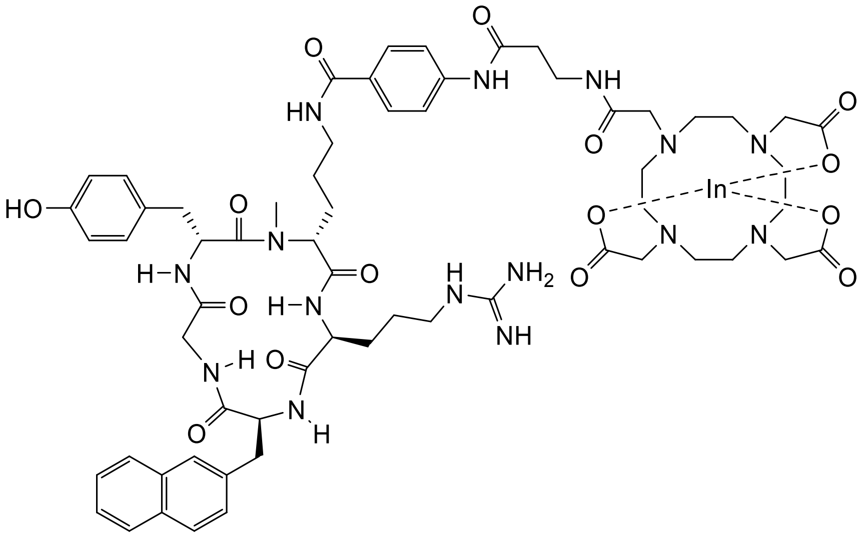


**30b**

**yorn'(Aba, betaAla, DOTA, In)RNalG**

*cyclo*(-D-Tyr-(α-methyl, δ-4-aminobenzoic acid, beta-alanine, DOTA, In)-D-Orn-R-Nal-G)

$C_{62}H_{80}InN_{15}O_{15}$

Exact Mass: 1389,5

Mol. Wt.: 1390,21

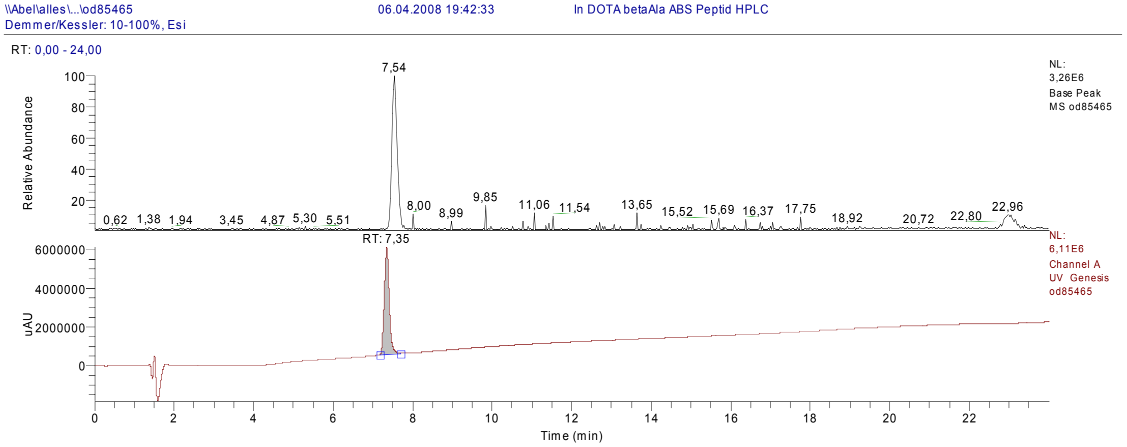


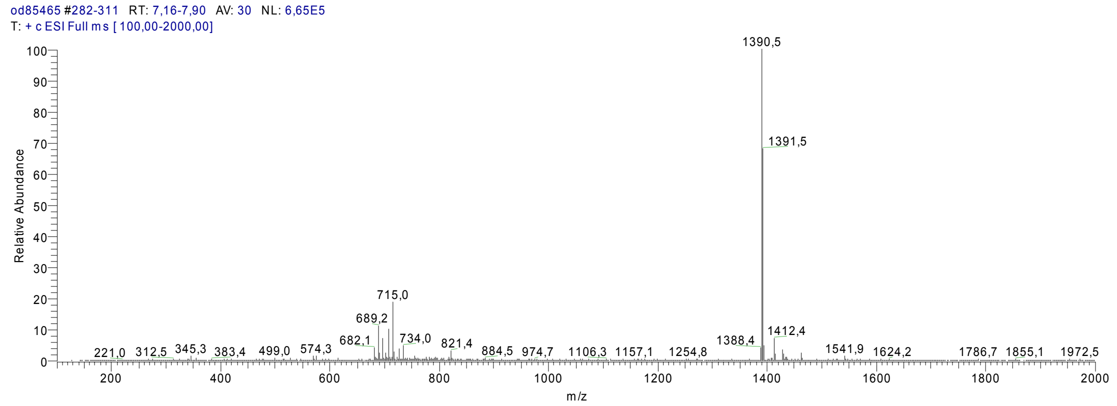

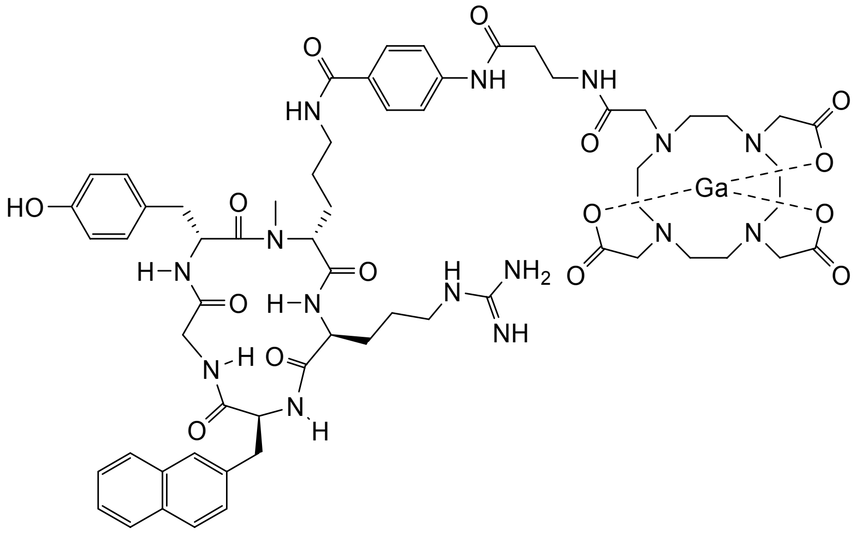


**30c**

**yorn'(Aba, betaAla, DOTA, Ga)RNalG**

*cyclo*(-D-Tyr-(α-methyl, δ-4-aminobenzoic acid, beta-alanine, DOTA, Ga)-D-Orn-R-Nal-G)

$C_{62}H_{80}GaN_{15}O_{15}$

Exact Mass: 1343,52

Mol. Wt.: 1345,11

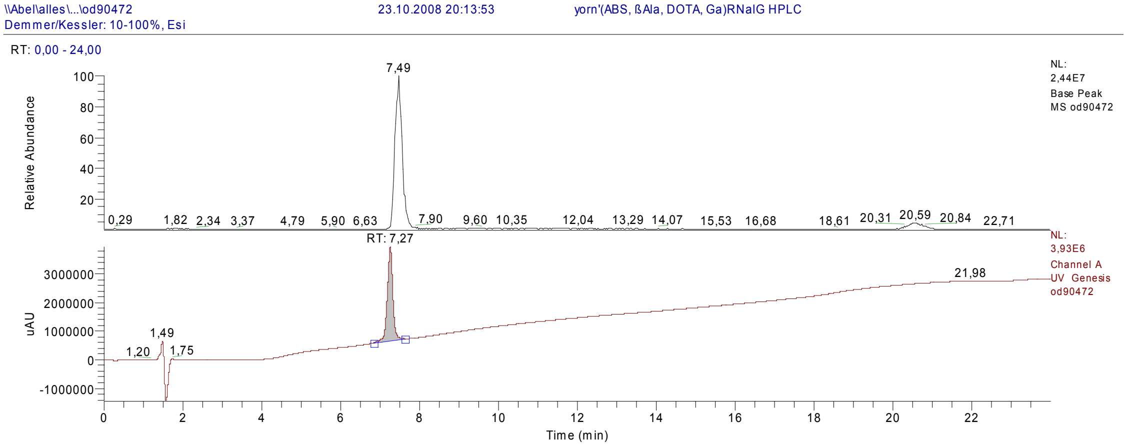


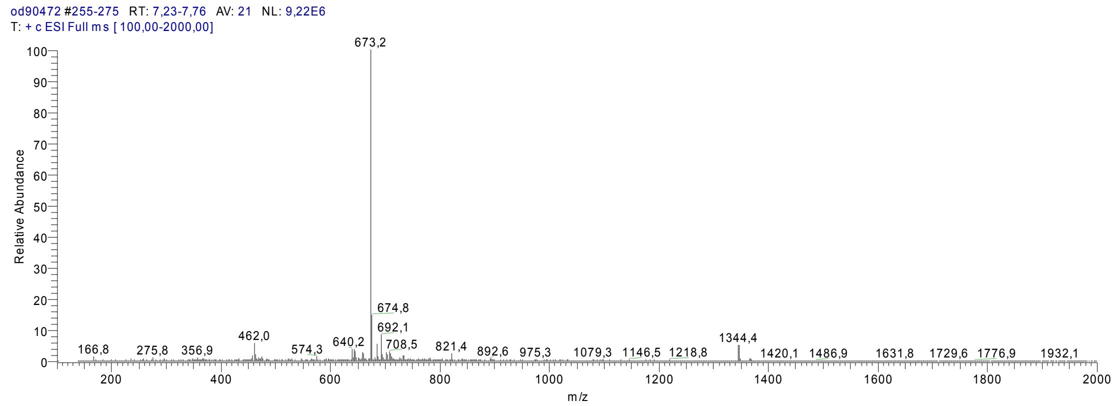

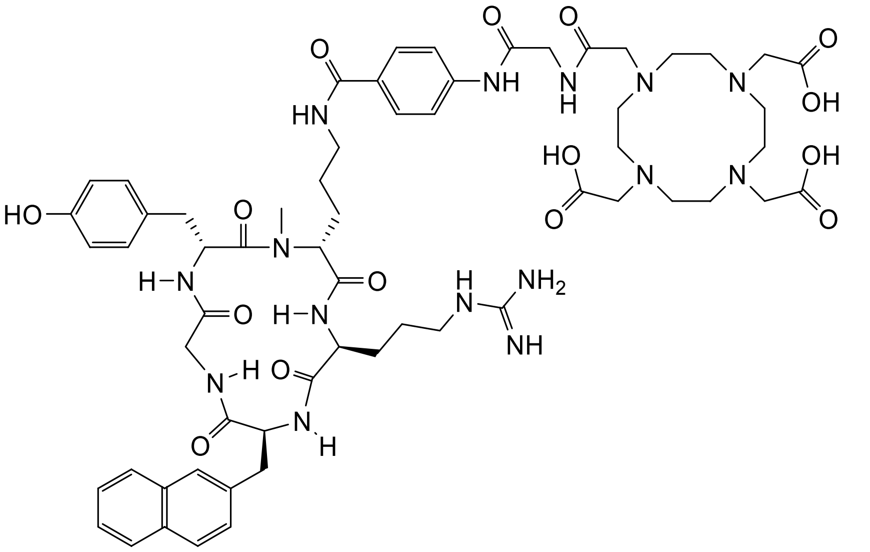


**31a**

**yorn'(Aba, G, DOTA)RNalG**

*cyclo*(-D-Tyr-(α-methyl, δ-4-aminobenzoic acid, G, DOTA)-D-Orn-R-Nal-G)

$C_{61}H_{81}N_{15}O_{15}$

Exact Mass: 1263,6

Mol. Wt.: 1264,39

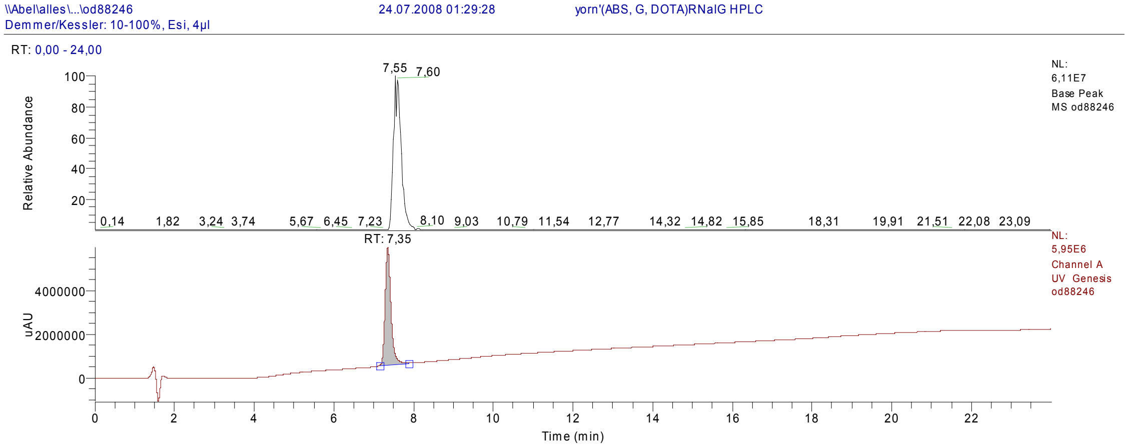


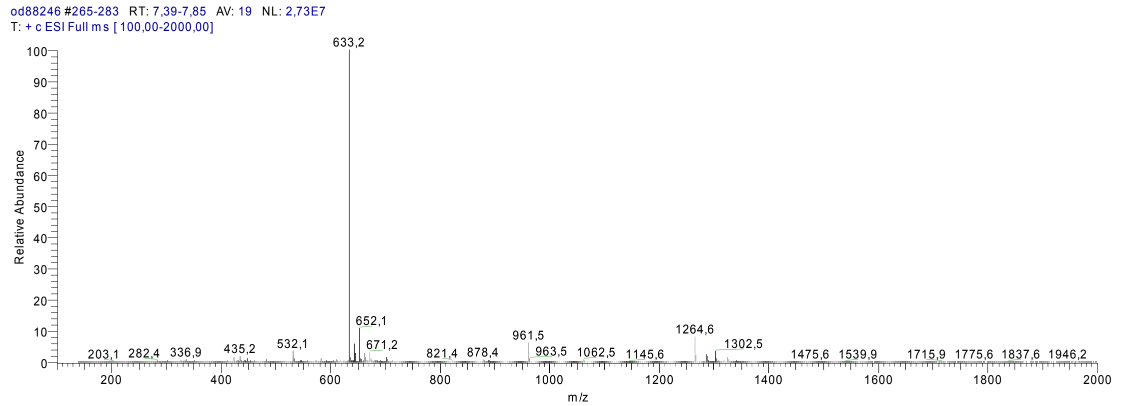

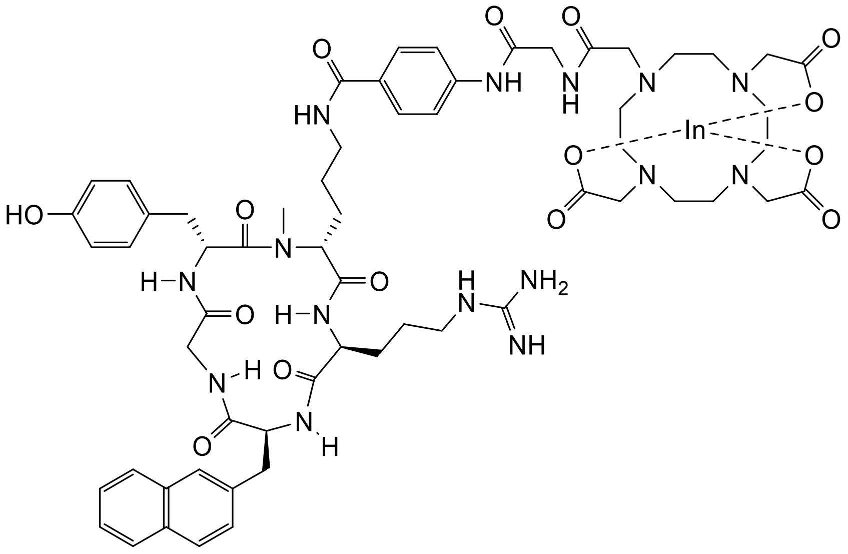

**31b**

**yorn'(Aba, G, DOTA, In)RNalG**

*cyclo*(-D-Tyr-(α-methyl, δ-4-aminobenzoic acid, G, DOTA, In)-D-Orn-R-Nal-G)

$C_{61}H_{78}InN_{15}O_{15}$

Exact Mass: 1375,48

Mol. Wt.: 1376,18

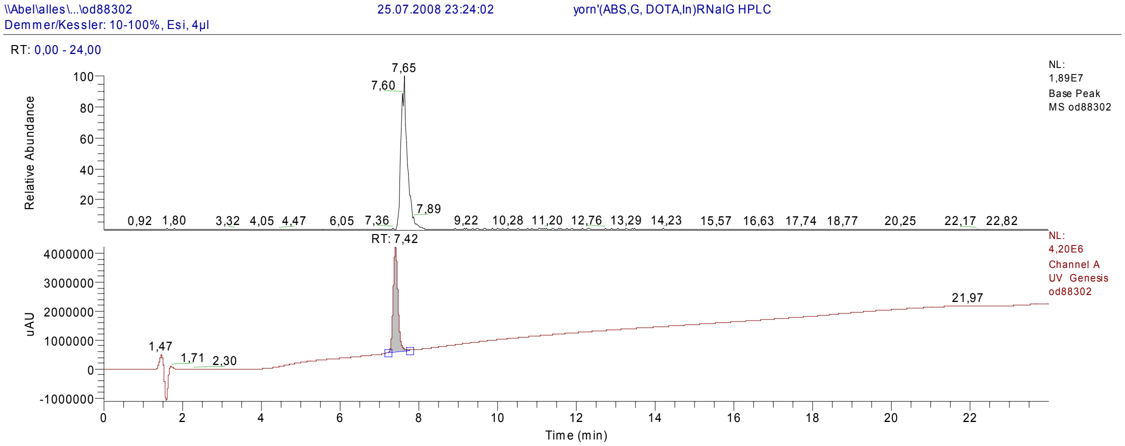


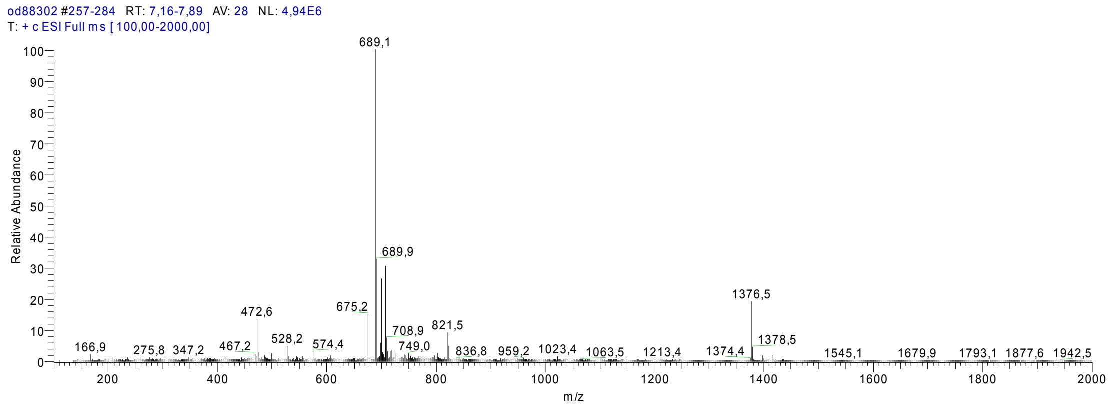

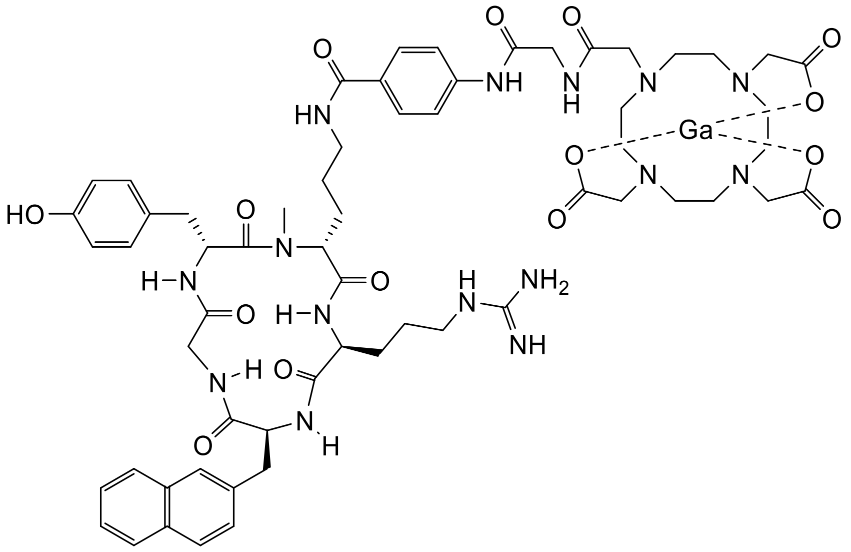

**31c**

**yorn'(Aba, G, DOTA, Ga)RNalG**

*cyclo*(-D-Tyr-(α-methyl, δ-4-aminobenzoic acid, G, DOTA, Ga)-D-Orn-R-Nal-G)

$C_{61}H_{78}GaN_{15}O_{15}$

Exact Mass: 1329,51

Mol. Wt.: 1331,09

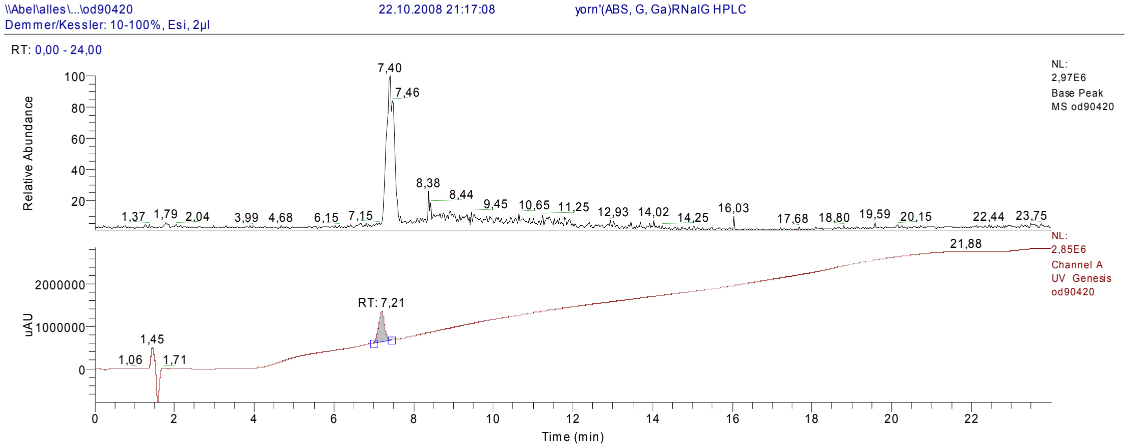


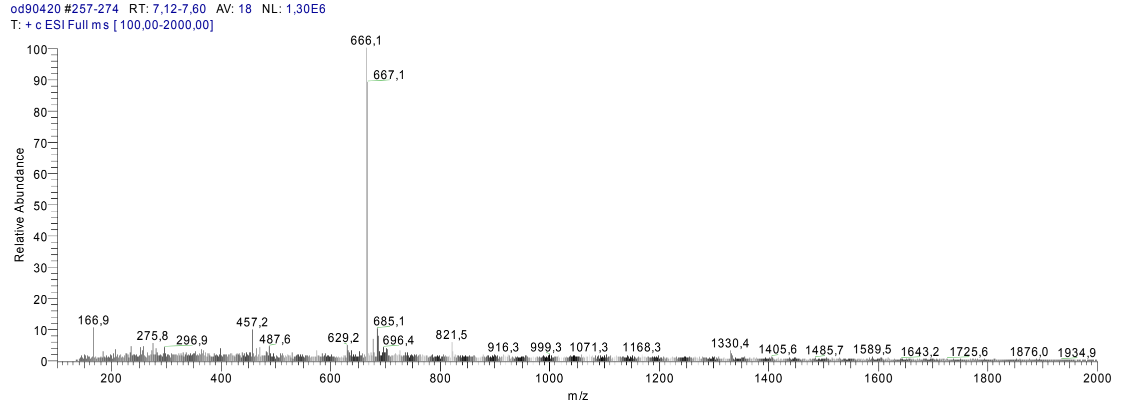

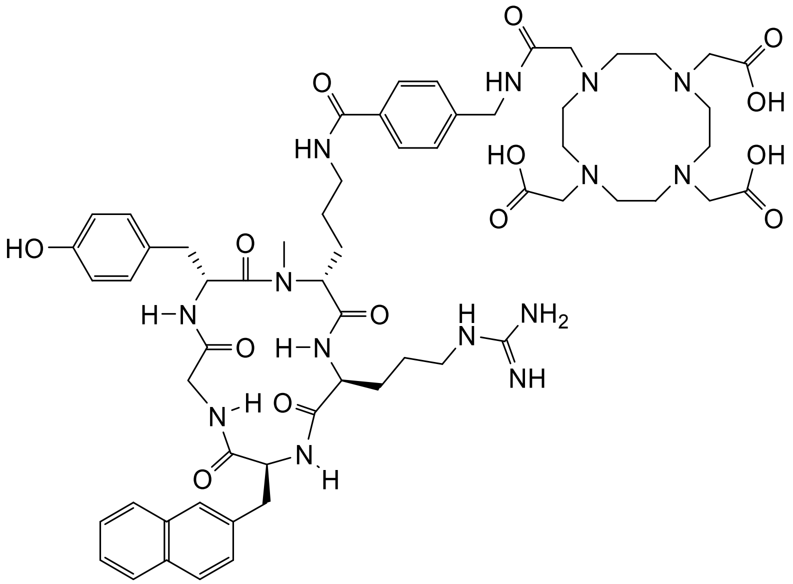


**2a**

**yorn'(Amba, DOTA)RNalG**

*cyclo*(-D-Tyr-(α-methyl, δ-4-(aminomethyl)benzoic acid, DOTA)-D-Orn-R-Nal-G)

$C_{60}H_{80}N_{14}O_{14}$

Exact Mass: 1220,6

Mol. Wt.: 1221,36

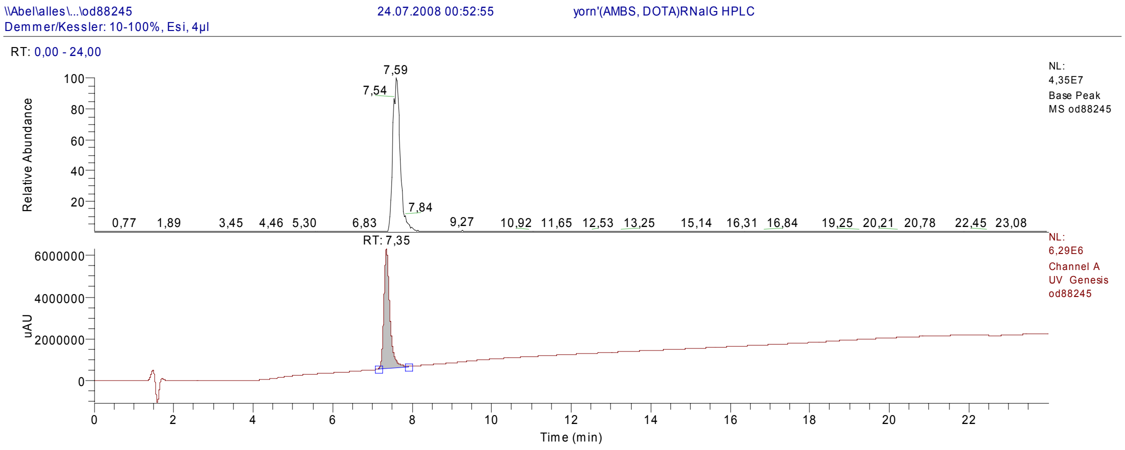


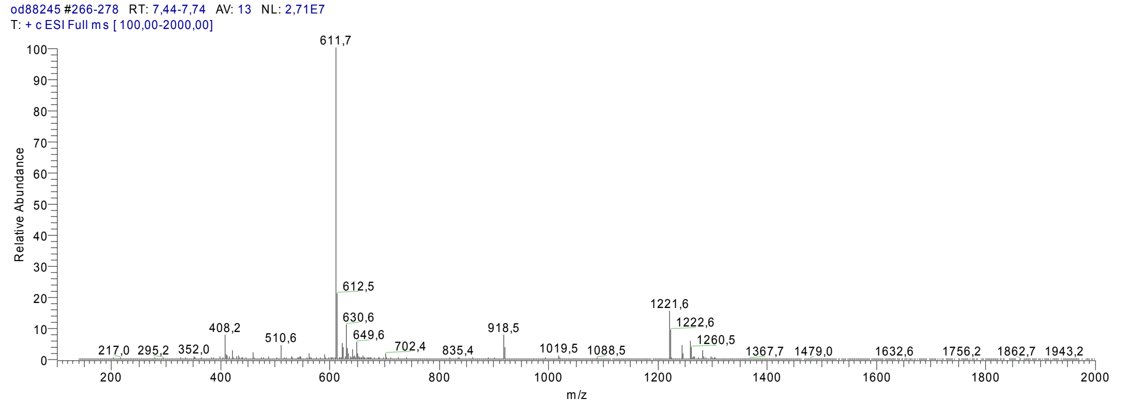

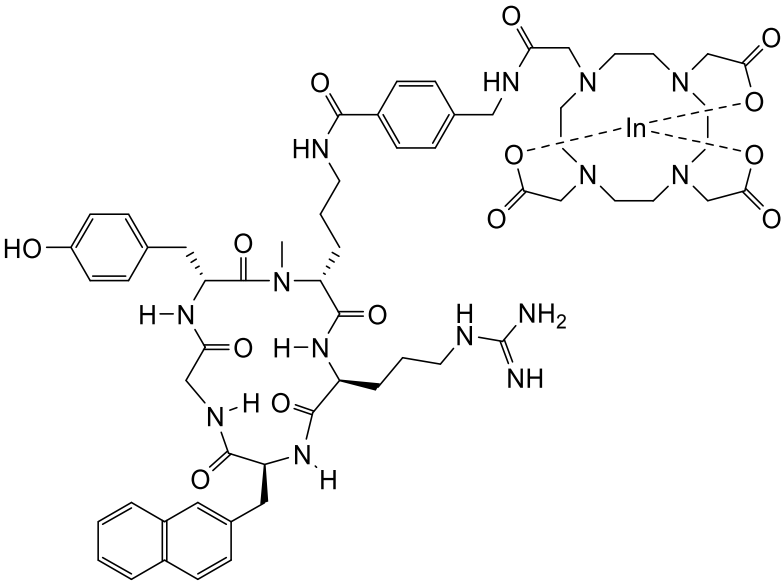


**2b**

**yorn'(Amba, DOTA, In)RNalG**

*cyclo*(-D-Tyr-(α-methyl, δ-4-(aminomethyl)benzoic acid, DOTA, In)-D-Orn-R-Nal-G)

$C_{60}H_{77}InN_{14}O_{14}$

Exact Mass: 1332,48

Mol. Wt.: 1333,16

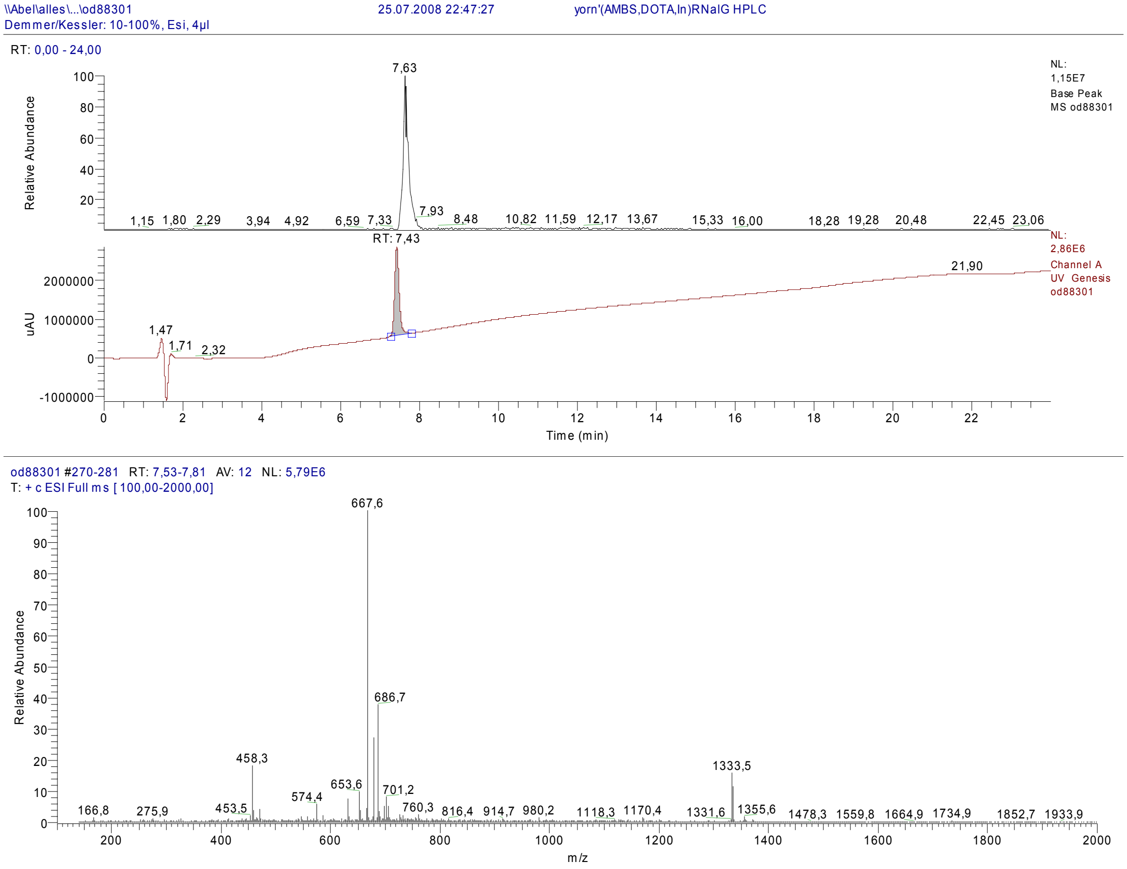

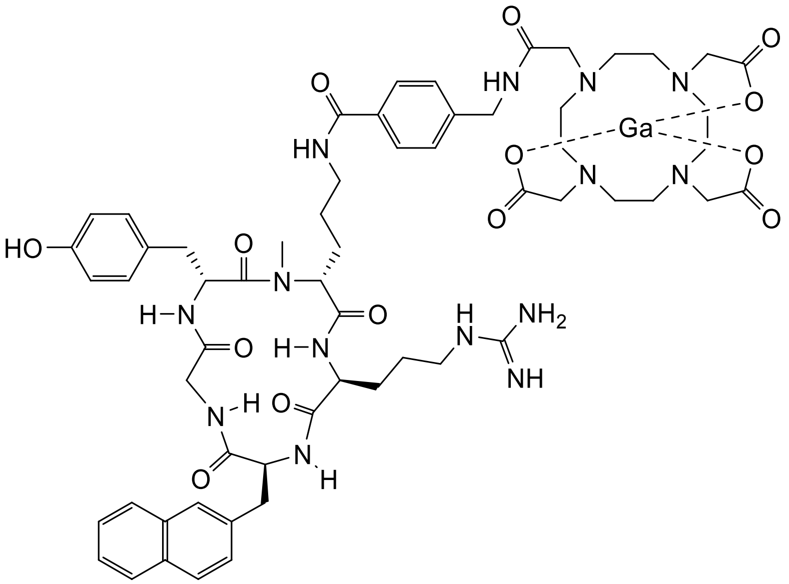

**2c**

**yorn'(Amba, DOTA, Ga)RNalG**

*cyclo*(-D-Tyr-(α-methyl, δ-4-(aminomethyl)benzoic acid, DOTA, Ga)-D-Orn-R-Nal-G)

$C_{60}H_{77}GaN_{14}O_{14}$

Exact Mass: 1286,5

Mol. Wt.: 1288,06

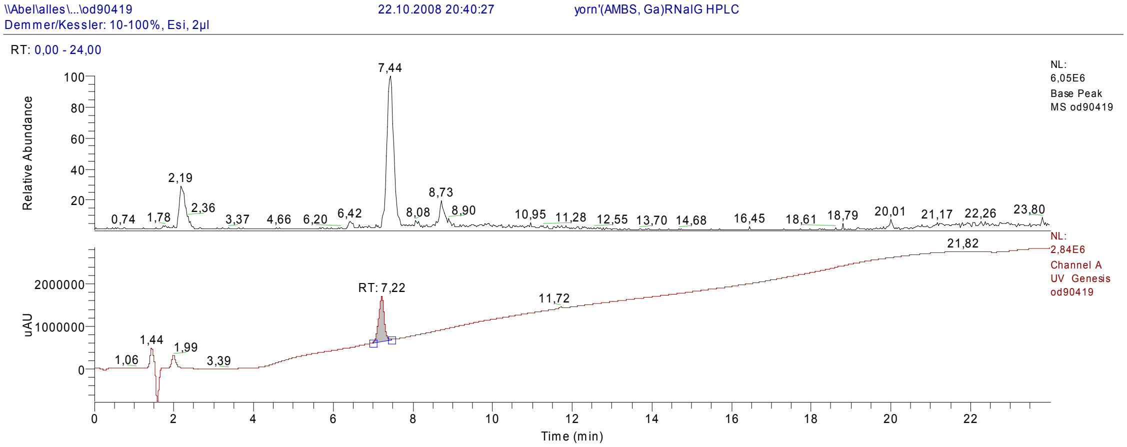


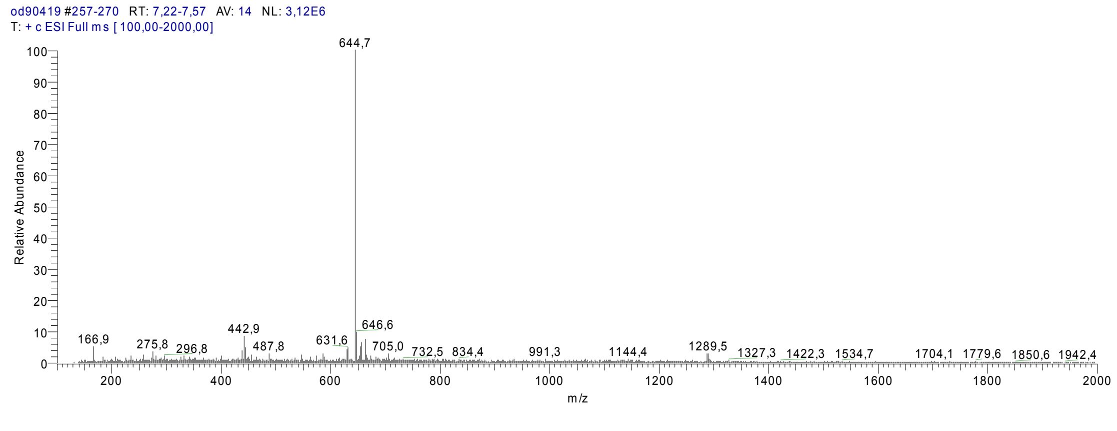

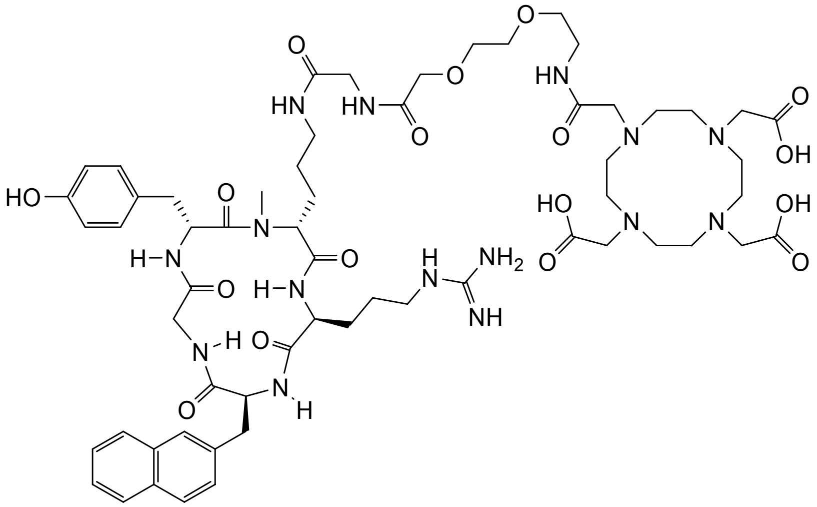


**33a**
**yorn'(G, Trigas, DOTA)RNalG**
*cyclo*(-D-Tyr-(α-methyl, δ-G, 2-(2-(2-aminoethoxy)ethoxy)acetic acid, DOTA)-D-Orn-R-Nal-G)
$C_{60}H_{87}N_{15}O_{17}$
Exact Mass: 1289,64
Mol. Wt.: 1290,42

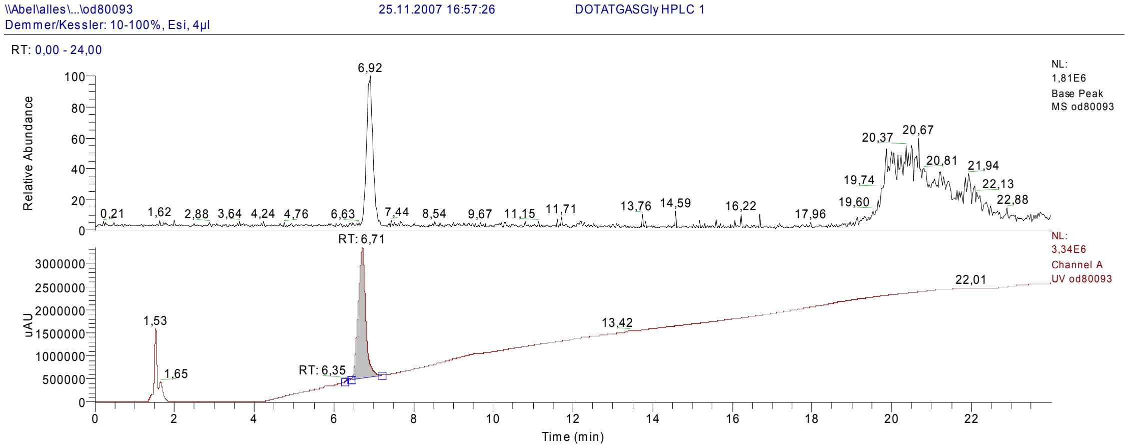


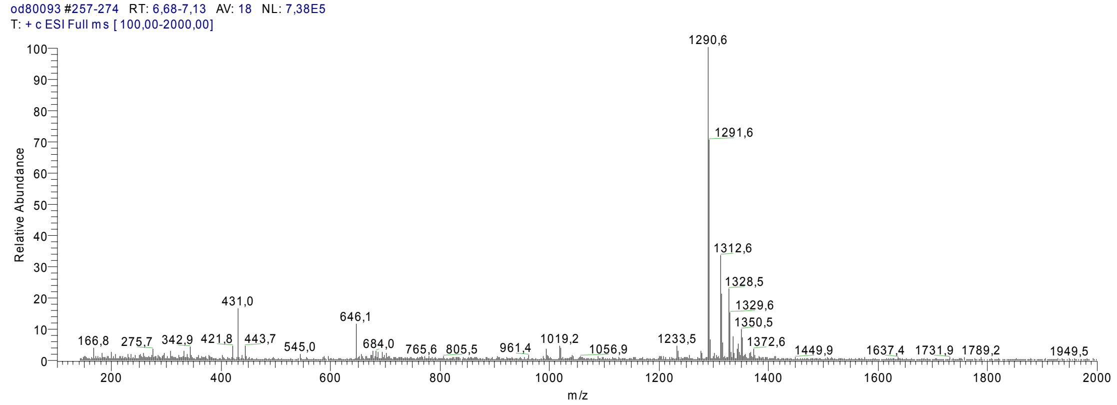

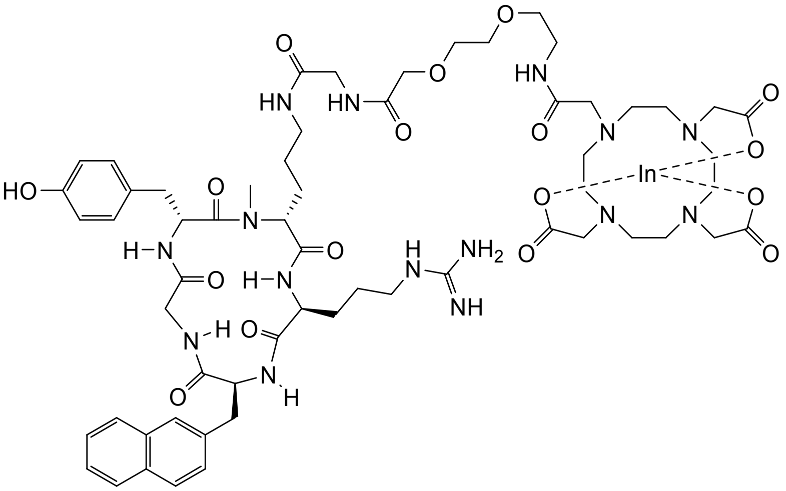

**33b**

**yorn'(G, Trigas, DOTA, In)RNalG**

*cyclo*(-D-Tyr-(α-methyl, δ-G, 2-(2-(2-aminoethoxy)ethoxy)acetic acid, DOTA, In)-D-Orn-R-Nal-G)

$C_{60}H_{84}InN_{15}O_{17}$

Exact Mass: 1401,52

Mol. Wt.: 1402,22

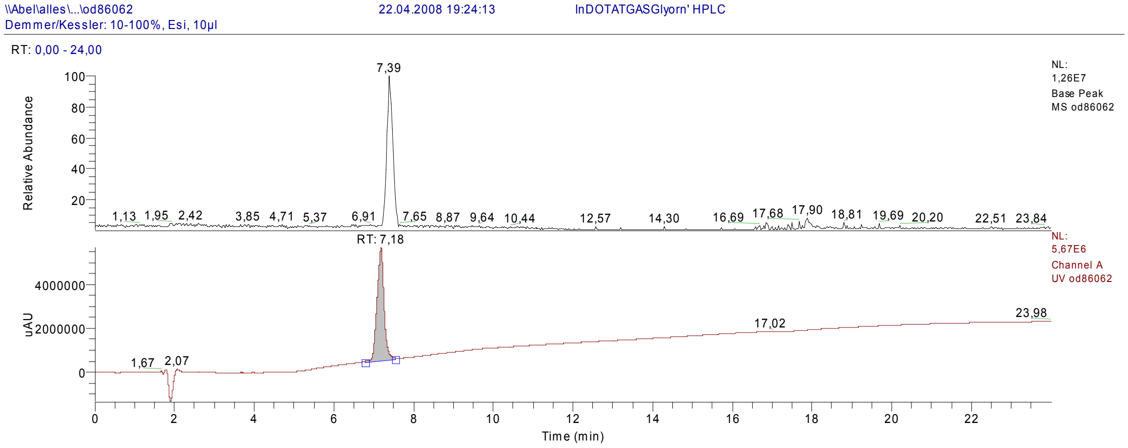


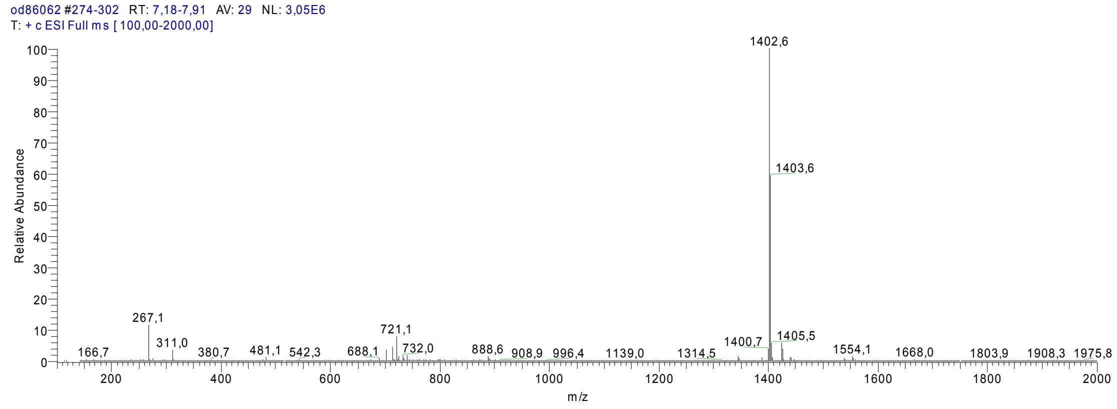

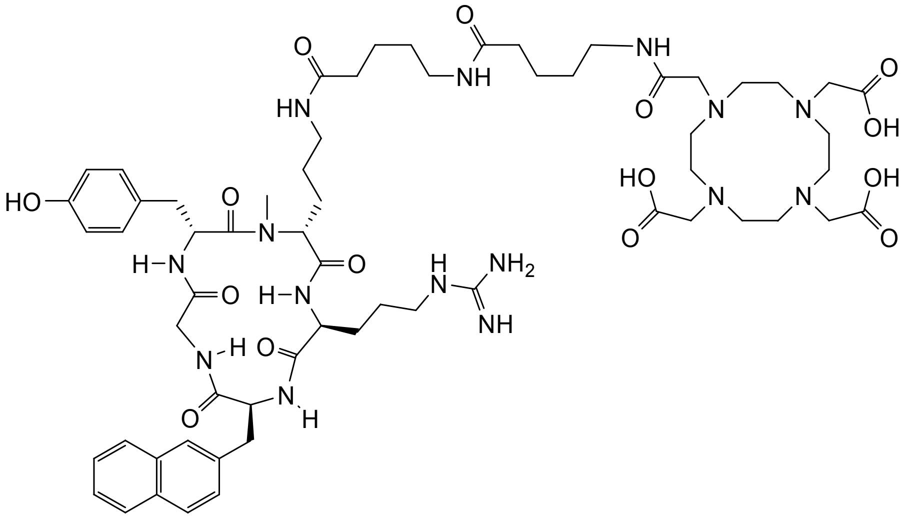


**34a**

**yorn'(Ava, Ava, DOTA)RNalG**

*cyclo*(-D-Tyr-(α-methyl, δ-5-aminovaleric acid, 5-aminovaleric acid, DOTA)-D-Orn-R-Nal-G)

$C_{62}H_{91}N_{15}O_{15}$

Exact Mass: 1285,68

Mol. Wt.: 1286,48

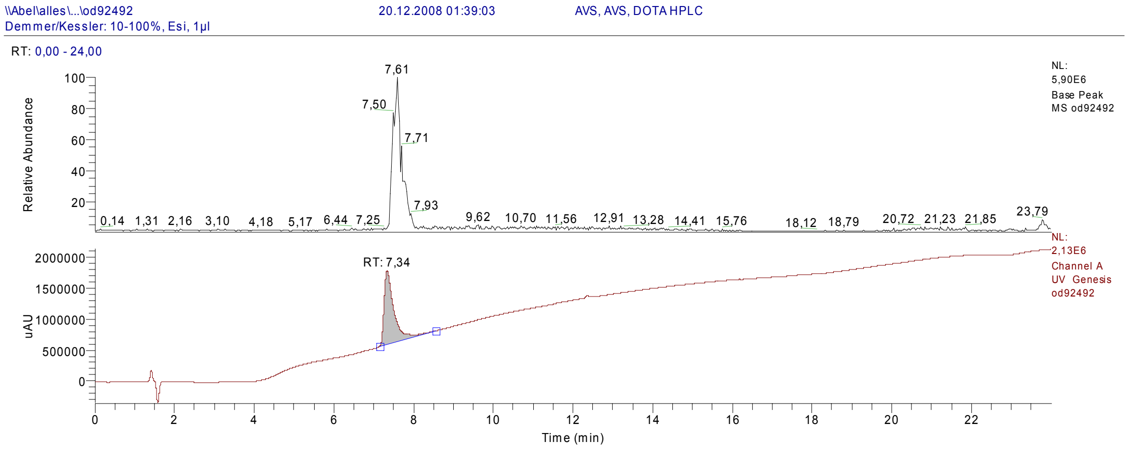


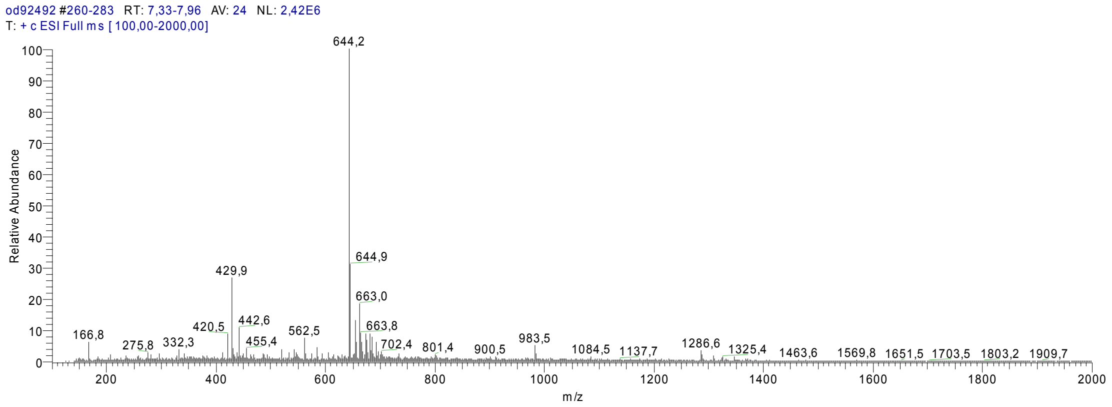

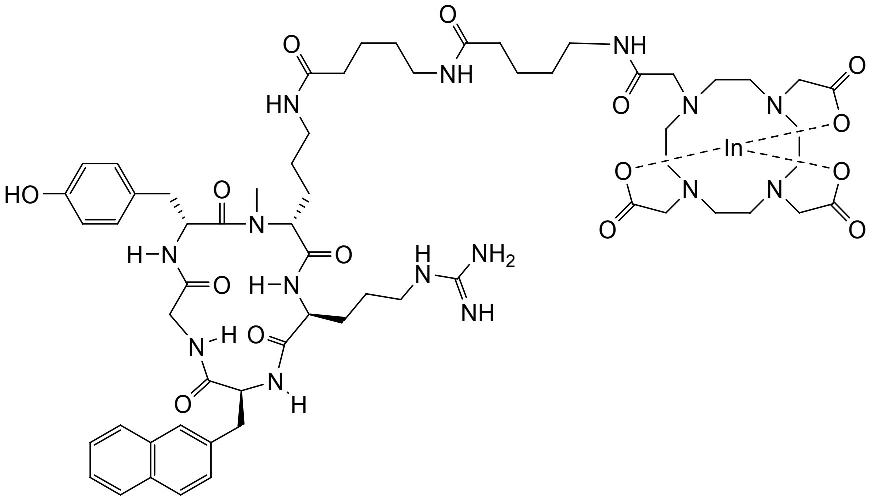


**34b**

**yorn'(Ava, Ava, DOTA, In)RNalG**

*cyclo*(-D-Tyr-(α-methyl, δ-5-aminovaleric acid, 5-aminovaleric acid, DOTA, In)-D-Orn-R-Nal-G)

$C_{62}H_{88}InN_{15}O_{15}$

Exact Mass: 1397,56

Mol. Wt.: 1398,27

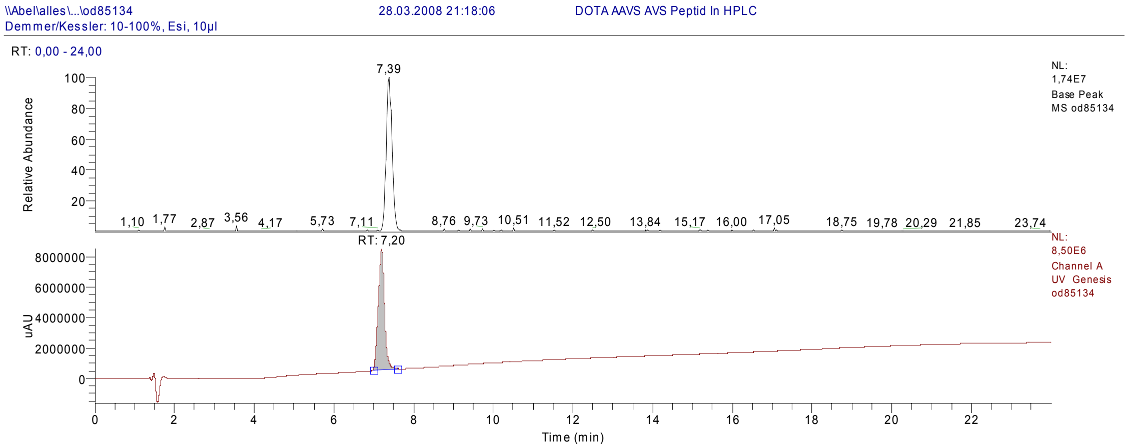


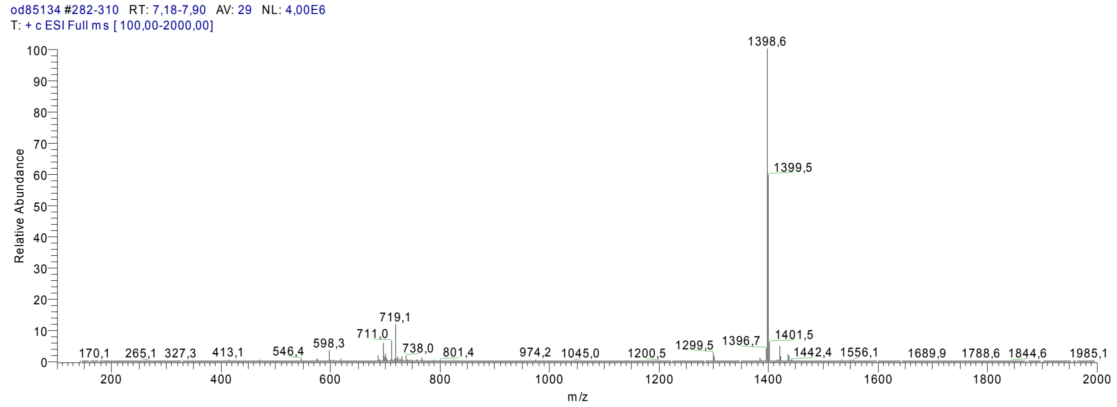

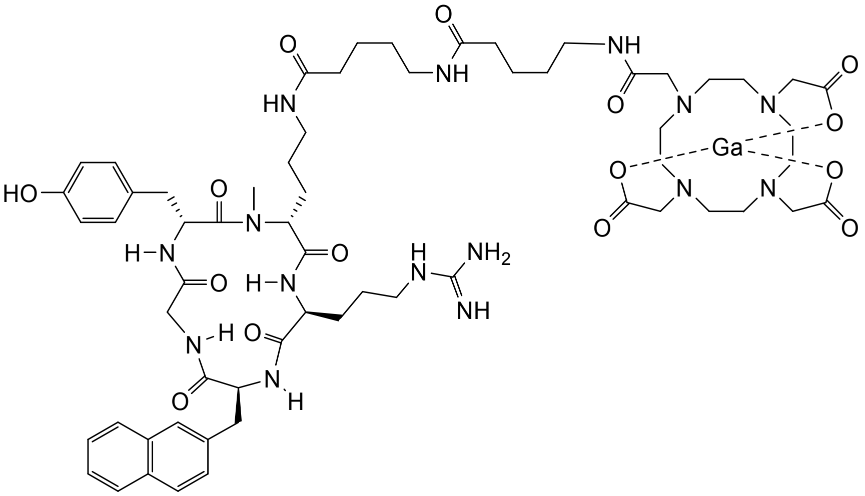


**34c**

**yorn'(Ava, Ava, DOTA, Ga)RNalG**

*cyclo*(-D-Tyr-(α-methyl, δ-5-aminovaleric acid, 5-aminovaleric acid, DOTA, Ga)-D-Orn-R-Nal-G)

$C_{62}H_{88}GaN_{15}O_{15}$

Exact Mass: 1351,58

Mol. Wt.: 1353,18

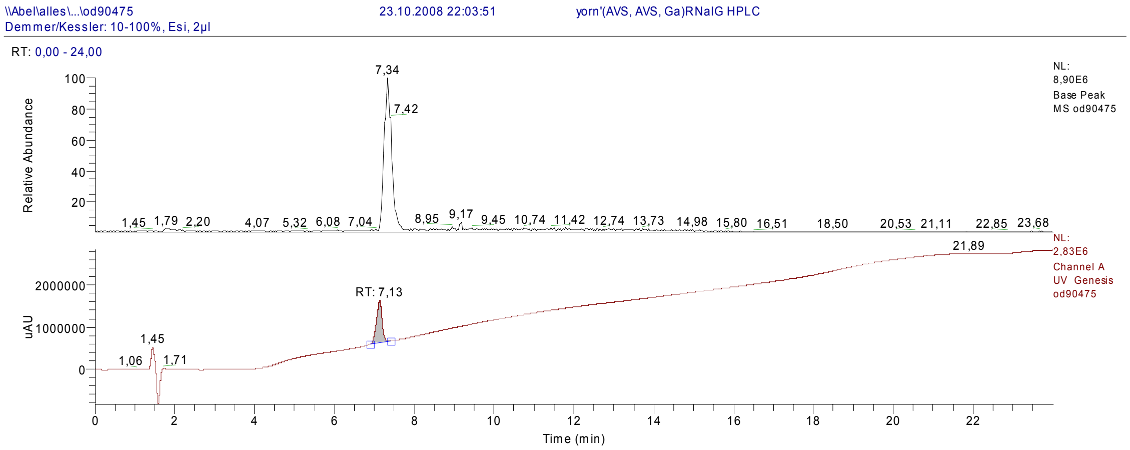


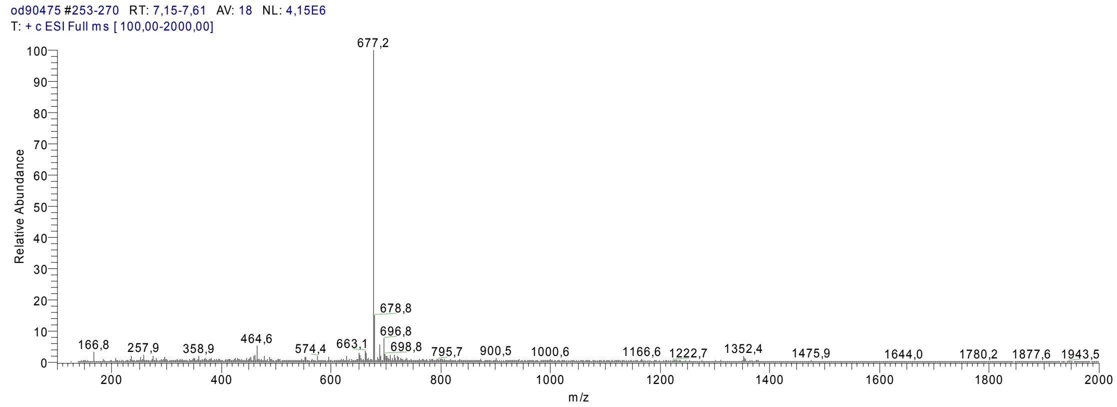

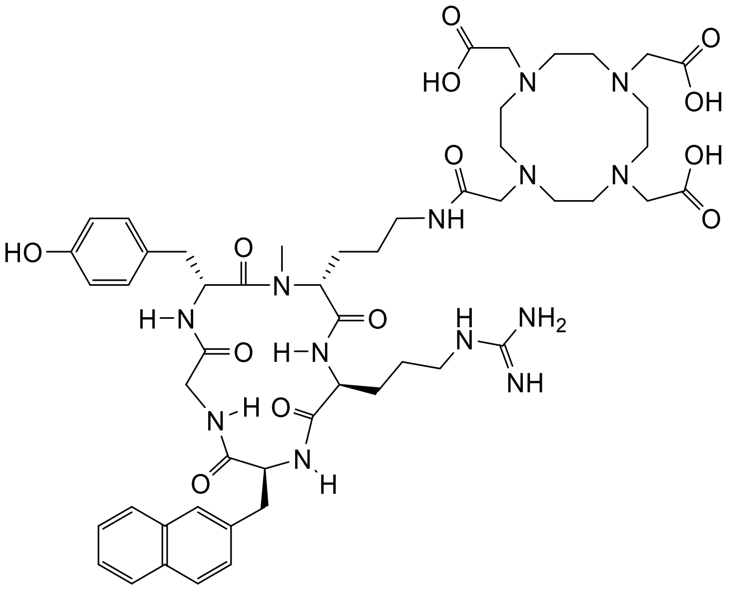

**35a**

**yorn'(DOTA)RNalG**

*cyclo*(-D-Tyr-(α-methyl, δ-DOTA)-D-Orn-R-Nal-G)

$C_{52}H_{73}N_{13}O_{13}$

Exact Mass: 1087,55

Mol. Wt.: 1088,22

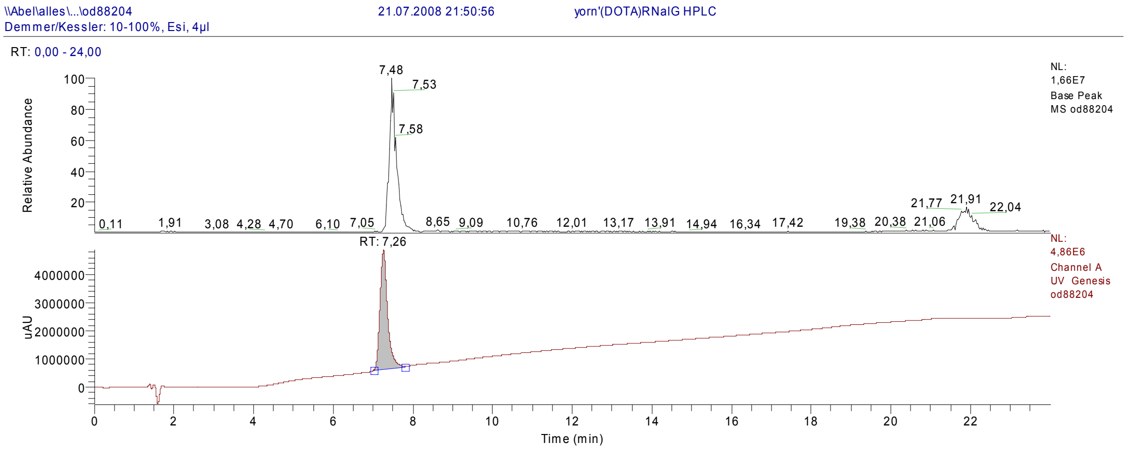


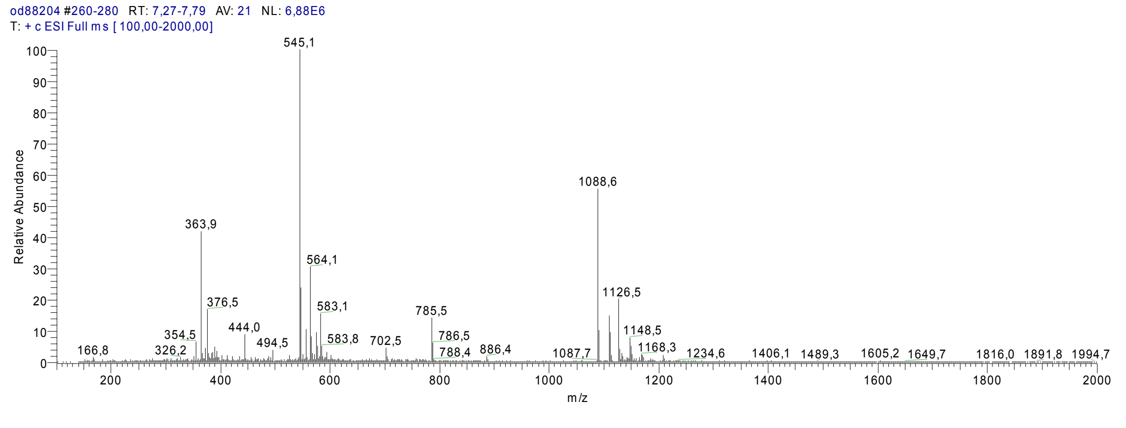

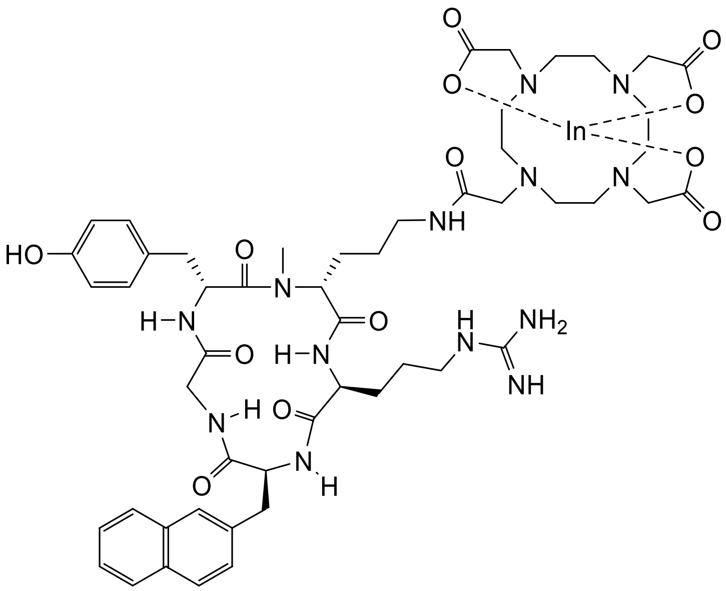

**35b**

**yorn'(DOTA, In)RNalG**

*cyclo*(-D-Tyr-(α-methyl, δ-DOTA-In)-D-Orn-R-Nal-G)

$C_{52}H_{70}InN_{13}O_{13}$

Exact Mass: 1199,43

Mol. Wt.: 1200,01

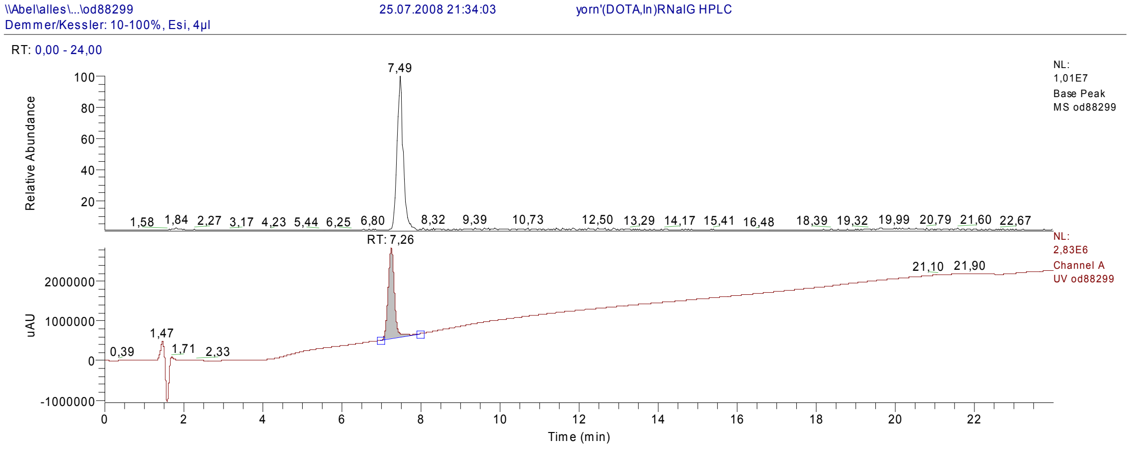


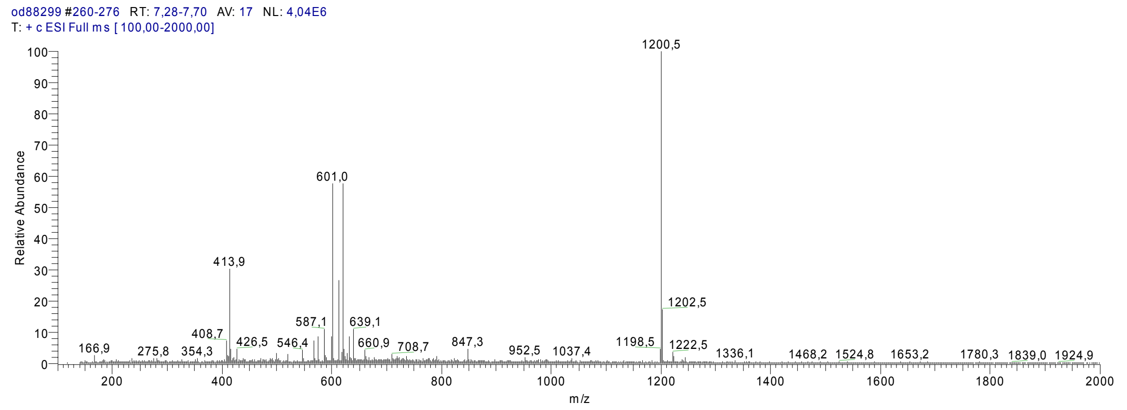

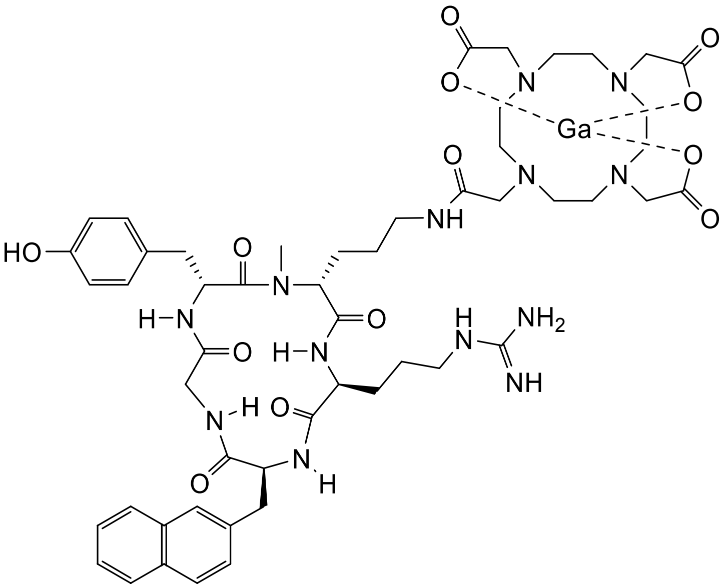


**35c**

**yorn'(DOTA, Ga)RNalG**

*cyclo*(-D-Tyr-(α-methyl, δ-DOTA-Ga)-D-Orn-R-Nal-G)

$C_{52}H_{70}GaN_{13}O_{13}$

Exact Mass: 1153,45

Mol. Wt.: 1154,91

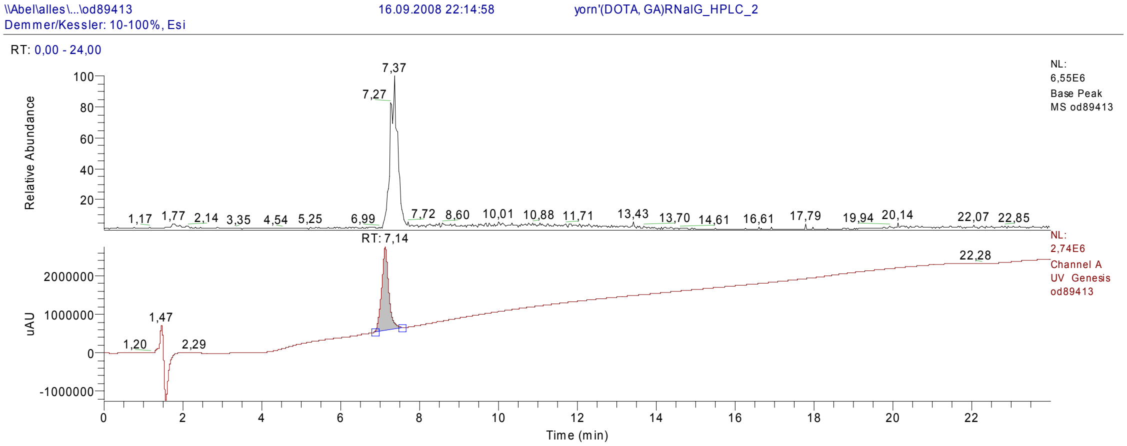


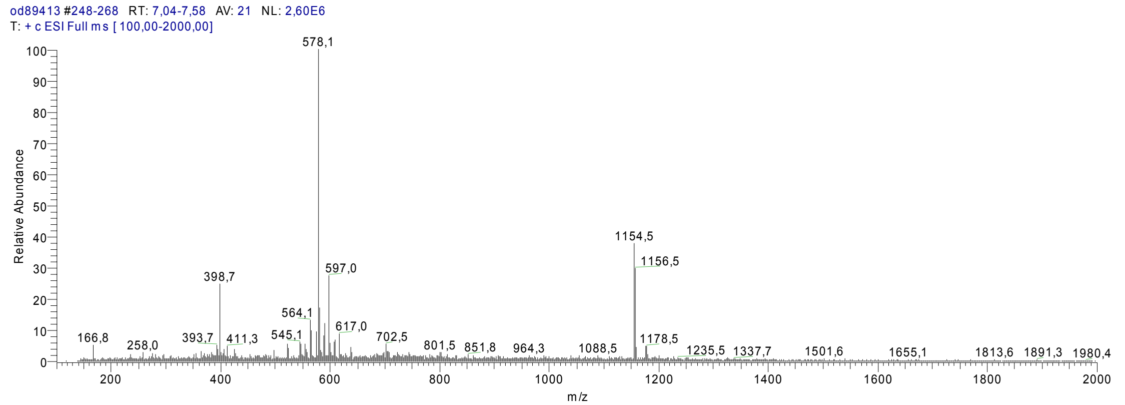

## Experimental Section

**General.** All commercially available chemical reagents were used without further purification. Technical solvents were distilled before use.

Trityl resins were purchased from *PepChem* and amino acid derivatives from *Iris Biotech GmbH*, *NovaBiochem*, *Merck*, *Bachem*, *Neosystem*, *Aldrich*, while all other chemicals were bought from *Aldrich*, *Fluka* und *Merck* if not stated otherwise.

NMP was obtained from *BASF* and used without further destillation. Dry solvents were purchased from *Aldrich*, *Fluka* and *Merck*. Dry dichloromethane was distilled from calciumhydride under argon and kept over 4 Å molecular sieve. Water for RP-HPLC was filtered through a 0.22 µm filter (Millipore, Millipak40).

RP-HPLC analyses were performed using an Amersham Pharmacia Biotech Äkta Basic 10F equipped with an Omnicrom YMC column (4.6 mm × 250 mm, 5 µm $C_{18}$, 1 mL/min). The eluent was a linear gradient from water (0.1% TFA) to acetonitrile (0.1% TFA) over 30 minutes and detection at 220 nm and 254 nm. The retention time ($R_t$) of the analytical RP-HPLC is given in minutes with the gradient in percentage of acetonitrile. Purities were determined at 220 nm with the Unicorn software package and are given relative to their starting compound.

Semi-preparative RP-HPLC was done on a *Beckman* System Gold equipped with high pressure module 125, UV-detector 166, and using an Omnicrom ODS-A C18 (120 Å, 5 µm, 250 mm × 20 mm) column in combination with the same solvents as stated above.

NMR spectra were recorded on a Bruker Avance 250 or Bruker DMX 500 at 298K. The chemical shifts are reported in ppm on the δ scale relative to the solvent signal. $^{13}$C-NMR-spectra were recorded using $^{1}$H-broad band decoupling. Pulse programs were taken from the Bruker library or written by members of our group. Samples were prepared in tubes with a diameter of 5 mm using 0.5 ml of deuterated solvent. The resulting spectra were processed on a PC workstation using Bruker TOPSPIN 1.3 and MestRe Nova software.

ESI mass spectra were recorded on a Finnigan LCQ in combination with a Agilent/HP 1100 RP-HPLC system using a *Omnicrom YMC* ODS-A C18 column (120 Å, 3 µm, 125 mm × 2 mm) with a flow rate of 0.2 mL/min. The eluent was a linear gradient from water to acetonitrile with 0.1% formic acid over 20 min with detection at 220 nm.

**Building blocks for solid phase peptide synthesis (SPPS).** 1,4,7-tris(*tert*-butoxycarbonylmethyl)-1,4,7,10-tetraazacyclododecane-10-acetic acid (tris(t-Bu)DOTA),[1] *N*-fluorenylmethoxycarbonyl-triethylenglycol-8-amino-1-acid (Fmoc-Trigas),[2] *N*-fluorenylmethyloxycarbonyl-*β-D*-galactopyranosylmethylamine (Fmoc-Gal-OH),[3] and 2-(1-(6-hydroxyhexylamino)ethylidene)-5,5-dimethylcyclohexane-1,3-dione (Dde-aminohexanol)[4] were synthesized according to literature.

**Peptide synthesis.** Standard Fmoc strategy with acid labile side chain protecting groups (t-Bu for Tyr and 2,2,4,6,7-pentamethyldihydrobenzofurane-5-sulfonyl (Pbf) for Arg) was employed to construct peptides on tritylchloridpolystyrene (TCP) Resin. Standard peptide bonds were built by coupling with *O*-(benzotriazol-1-yl)-*N,N,N',N'*-tetramethyluronium tetrafluoroborate (TBTU) and addition of *N*-hydroxybenzotriazole (HOBt) to suppress racemization. *N*-alkylated amines were acylated using 2-(7-aza-1H-benzotriazole-1-yl)-1,1,3,3-tetramethyluronium hexafluorophosphate (HATU) with 1-hydroxy-7-azabenzotriazole (HOAt) as racemization suppressant. HATU was also used to acylate anilines and to attach tris(t-Bu)DOTA. Gly was chosen as C-terminal residue to avoid racemization in the cyclization step and at the same time raise its yields by turn preformation of the N-terminal D-amino acid.[5-7] In the case of peptides with spacers having free amines in the side chain tyrosine was placed at their C-terminus to avoid simultaneous deprotection of the Fmoc group in the backbone and the spacer. In this case the Alloc protected N-terminus at ornithine was orthogonally deprotected, the peptide cleaved and cyclized, and the Fmoc group in the elongated side chain deprotected in solution.
*N*-alkylation was achieved via the Fukuyama-Mitsunobu reaction by treating Ns-protected amines with an alcohol under typical Mitsunobu conditions (diisopropylazodicarboxylate (DIAD) and triphenylphosphine).[4] Ns was cleaved by treatment with 1,8-diazabicyclo[5.4.0]undec-7-ene (DBU) and 2-mercaptoethanol to yield the secondary amine.
The peptides were cleaved from the resin with 20% 1,1,1,3,3,3-hexafluoroisopropanol (HFIP) in DCM and cyclized with DPPA and $NaHCO_3$ in DMF. In the case of compounds containing Dde and Ns protecting groups these were cleaved with 2% hydrazine in DMF or with DBU and 2-mercaptoethanol, respectively. Final deprotection of acid labile groups was done in 95% trifluoroacetic acid (TFA) containing 2.5% $H_2O$ and 2.5% triisopropylsilane (TIPS) before RP-HPLC purification. ESI-MS was used to identify the peptides and the purity determined by analytical RP-HPLC was better than 95%.

**General amine protection procedure.** To a 0.2 M solution of amino acid and $Na_2CO_3$ (0.5 M) the same volume of a 0.2 M reagent solution in THF was added and stirred at RT for 1 h. The THF was evaporated under reduced pressure, the aqueous phase washed once with diethylether and acidified with conc. HCl to pH 1 and the product extracted with EtOAc. The combined organic layers were dried ($Na_2SO_4$), filtered, concentrated and dried in vacuo.

**Boc deprotection.** 3-8 mmol of Boc protected amino acid were dissolved in 10 mL DCM and 5 mL of TFA added slowly. The solution was stirred at RT for 45 min and the solvent evaporated in vacuo to yield the crude product ready for reprotection.

**Loading of Tritylchloridpolystyrene (TCP) Resin.** Peptide synthesis was carried out using TCP-resin (0.9 mmol/g) following standard Fmoc-strategy. Fmoc-Xaa-OH (1.2 eq.) were attached to the TCP resin with DIEA (2.5 eq.) in anhydrous DCM (0.8 mL/g resin) at room temperature for 1 h. The remaining trityl chloride groups were capped by addition of 1 mL/g(resin) of a solution of MeOH, DIEA (5:1; v:v) for 15 min. The resin was filtered and washed 5 times with DCM and 3 times with MeOH. The loading capacity was determined by weight after drying the resin under vacuum and ranged from 0.4-0.9 mmol/g.

**On-Resin Fmoc Deprotection.** The resin-bound Fmoc peptide was treated with 20% piperidine in NMP (v/v) for 10 min and a second time for 5 min. The resin was washed 5 times with NMP.

**TBTU/HOBt Coupling.** A solution of Fmoc-Xaa-OH (2 eq.), TBTU (2 eq.), HOBt (2 eq.), DIEA (5.2 eq.) in NMP (1 mL/g resin) was added to the resin-bound free amine peptide and shaken for 60 min at room temperature and washed 5 times with NMP.

**Ns Protection.** A solution of Ns-Cl (5 eq.) and collidine (10 eq.) in NMP (1 mL/g resin) was added to the resin-bound free amine peptide and shaken for 15 min at room temperature. The resin was washed 3 times with NMP and 3 times with dry THF.

***N*-Alkylation under Mitsunobu Conditions.** A solution of triphenylphosphine (5 eq.), DIAD (5eq.) and ROH (10 eq.) in dry THF (1 mL/g resin) was added to the resin-bound Ns protected peptides and shaken at room temperature (methylation: 10 min; alkylation with Dde-Aminohexanol: 2 times 30 min). The resin was filtered off, and washed 3 times with dry THF and 3 times with NMP.

**On-resin attachment of 2,4-dinitrobenzene to a primary amine.** The resin with the free amine was shaken at RT with DIEA (4 eq.) and 1-fluoro-2,4-dinitrobenzene (10 eq.) in DCM (1 mL/g resin) for 20 min and subsequently washed 5 times with DCM.

**On-Resin Ns Deprotection.** For Ns deprotection, the resin-bound Ns-peptides were stirred in a solution of mercaptoethanol (10 eq.) and DBU (5 eq.) in NMP (1 mL/g resin) for 5 minutes. The deprotection procedure was repeated one more time and the resin was washed 5 times with NMP.

**HATU/HOAt Coupling.** A solution of Fmoc-Xaa-OH (2 eq.), HATU (2 eq.), HOAt (2 eq.), DIEA (4 eq.) in NMP (1 mL/g resin) was added to the resin-bound peptides and shaken for 3 h at room temperature and washed 5 times with NMP.

**Alloc Deprotection.** $Pd(PPh_3)_4$ (0.125 eq.) in dry DCM (0.5 mL/g resin) was added to the resin-bound Alloc peptide followed by an addition of phenylsilan in

dry DCM (0.5 mL/g resin) and shaken for 1 h. The resin was washed 5 times with DCM.

**Peptide Cleavage.** For complete cleavage from the resin the peptides were treated three times with a solution of DCM and HFIP (4:1; v:v) at room temperature for half an hour and the solvent evaporated under reduced pressure.

**Cyclization.** To a solution of peptide in DMF (1 mM peptide concentration) and $NaHCO_3$ (5 eq.) DPPA (3 eq.) was added at RT and stirred over night or until no linear peptide could be observed by ESI-MS. The solvent was evaporated to a small volume under reduced pressure and the peptides precipitated in saturated NaCl solution and washed two times in HPLC grade water.

**Ns Deprotection in Solution.** For Ns deprotection cyclized peptides were treated with a solution of mercaptoethanol (10 eq.) and DBU (5 eq.) in 2.5 mL DMF for 30 min and precipitated in saturated NaCl solution and washed two times in HPLC grade water.

**Dde Deprotection in Solution.** For Dde-group deprotection, the peptides were stirred in a solution of 2% hydrazine in DMF for 15-30 min and precipitated in saturated NaCl solution and washed two times in HPLC grade water.

**Fmoc Deprotection in Solution.** The cyclic peptide was treated with 2.5 mL 20% piperidine in DMF (v/v) for 30 min and precipitated in saturated NaCl solution and washed two times in HPLC grade water.

**Removal of Acid Labile Side Chain Protecting Groups.** Cyclized peptides were stirred in a solution of TFA, water and TIPS (95:2.5:2.5; v:v:v) at room temperature for 1 h or until no more protected peptide could be observed by ESI-MS and precipitated in diethylether and washed two more times.

**Chelation of In with DOTA Ligands.** DOTA ligands were dissolved in 5 M ammoniumacetate (0.5 mL; pH 4.5) and treated with $InCl_3$ (5 eq.) dissolved in 5 M ammoniumacetate (0.05 mL). After 15 min of stirring at RT the solution was subjected to HPLC purification.

**Chelation of Ga with DOTA Ligands.** DOTA ligands were dissolved in 0.01 M ammoniumacetate (0.5 mL; pH 4.5) and treated with $Ga(NO_3)_3$ (10 eq.) dissolved in 0.01 M ammoniumacetate (0.05 mL) resulting in a final pH of 3. After 2-4 h of stirring at RT the solution was subjected to HPLC purification.

**$N^{\alpha}$-Alloc-$N^{\delta}$-Boc-L-ornithine.** $N^{\delta}$-Boc-L-ornithine (1.00 g, 4.3 mmol) was protected with allyl chloroformate (0.46 ml, 4.3 mmol) and gave a colourless, sticky oil as sufficiently pure product (1.20 g, 90%). $^1$H NMR (250 MHz, DMSO-$d_6$): δ 12.52 (s, 1H), 7.49 (d, 1H), 6.78 (t, 1H), 5.91 (br m, 1H), 5.30 (dd, 1 H), 5.19 (dd, 1 H), 4.48 (m, 2H), 3.91 (br m, 1H), 2.91 (m, 2H), 1.81-1.40 (br m, 4H),

1.38 (s, 9H). $^{13}C$ NMR (63 MHz, DMSO-$d_6$): 174.4, 156.5, 156.1, 134.1, 117.4, 77.9, 65.1, 60.2, 54.1, 28.8, 26.7, 14.6. $R_t$ (10-100%): 16.7 min. *m/z* calcd for $C_{14}H_{24}N_2O_6$: 316.16; found 339.3 [M + $Na^+$].

***N*$^{\alpha}$-Alloc-*N*$^{\delta}$-Boc-D-ornithine.** *N*$^{\delta}$-Boc-D-ornithine (0.49 g, 2.1 mmol) was protected with allyl chloroformate (0.22 ml, 2.1 mmol) and gave a slightly yellow, sticky oil as sufficiently pure product (0.53 g, 80%). $^1H$ NMR (250 MHz, DMSO-$d_6$): δ 12.52 (s, 1H), 7.49 (d, 1H), 6.78 (t, 1H), 5.91 (br m, 1H), 5.30 (dd, 1 H), 5.19 (dd, 1 H), 4.48 (m, 2H), 3.91 (br m, 1H), 2.91 (m, 2H), 1.81-1.40 (br m, 4H), 1.38 (s, 9H). $^{13}C$ NMR (63 MHz, DMSO-$d_6$): 174.4, 156.5, 156.1, 134.1, 117.4, 77.9, 65.1, 60.2, 54.1, 28.8, 26.7, 14.6. $R_t$ (10-100%): 16.7 min. *m/z* calcd for $C_{14}H_{24}N_2O_6$: 316.16; found 339.3 [M + $Na^+$].

***N*$^{\alpha}$-Alloc-*N*$^{\varepsilon}$-Boc-L-lysine.** *N*$^{\varepsilon}$-Boc-L-lysine (0.49 g, 2.0 mmol) was protected with allyl chloroformate (0.21 mL, 2.0 mmol) and gave a slightly yellow, sticky oil as sufficiently pure product (0.57 g, 82%). $^1H$ NMR (250 MHz, DMSO-$d_6$): δ 12.48 (s, 1H), 7.44 (d, 1H), 6.74 (br s, 1H), 5.89 (br m, 1H), 5.23 (dd, 2H), 4.47 (d, 2H), 3.89 (m, 2H), 2.87 (m, 2H), 1.62 (m, 2H), 1.37 (br m, 13H). $^{13}C$ NMR (63 MHz, DMSO-$d_6$): 174.4, 156.4, 156.0, 134.0, 117.4, 77.8, 64.9, 54.2, 30.9, 28.7, 23.4, 14.5. $R_t$ (10-100%): 17.7 min. *m/z* calcd for $C_{15}H_{26}N_2O_6$: 330.18; found 353.3 [M + $Na^+$].

***N*$^{\alpha}$-Alloc-*N*$^{\delta}$-Fmoc-L-ornithine.** *N*$^{\alpha}$-Alloc-*N*$^{\delta}$-Boc L-ornithine (1.20 g, 3.87 mmol) was subjected to Boc deprotection and subsequently reprotected with Fmoc-OSu (1.31 g, 3.87 mmol) and gave a white foam as sufficiently pure product (1.12 g, 66%). $^1H$ NMR (500 MHz, DMSO-$d_6$): δ 12.5 (s, 1H), 7.9 (d, 2H), 7.7 (d, 2H), 7.5 (d, 1H), 7.4 (t, 2H), 7.32 (t, 2H), 7.28 (m, 1H), 5.9 (m, 1H), 5.3 (d, 1H), 5.2 (d, 1H), 4.5 (d, 2H), 4.3 (d, 2H), 4.2 (t, 1H), 3.9 (m, 1H), 3.0 (d, 2H), 1.7 (m, 1H), 1.5 (m, 3H). $^{13}C$ NMR (125 MHz, DMSO-$d_6$): 174, 156.0, 155.9, 144, 141, 133, 128, 127.0, 126.9, 125.1, 125.0, 120.1, 119.9, 65, 64, 53.54, 53.50, 47, 28, 26. $R_t$ (10-100%): 21.9 min. *m/z* calcd for $C_{24}H_{26}N_2O_6$: 438.18; found 439.3 [M + $H^+$].

***N*$^{\alpha}$-Alloc-*N*$^{\delta}$-Fmoc-D-ornithine.** *N*$^{\alpha}$-Alloc-*N*$^{\delta}$-Boc-D-ornithine (0.36 g, 1.68 mmol) was subjected to Boc deprotection and subsequently reprotected with Fmoc-OSu (0.567 g, 1.68 mmol) and gave a white foam as sufficiently pure product (0.61 g, 89%). $^1H$ NMR (500 MHz, DMSO-$d_6$): δ 12.5 (s, 1H), 7.9 (d, 2H), 7.7 (d, 2H), 7.5 (d, 1H), 7.4 (t, 2H), 7.32 (t, 2H), 7.28 (m, 1H), 5.9 (m, 1H), 5.3 (d, 1H), 5.2 (d, 1H), 4.5 (d, 2H), 4.3 (d, 2H), 4.2 (t, 1H), 3.9 (m, 1H), 3.0 (d, 2H), 1.7 (m, 1H), 1.5 (m, 3H). $^{13}C$ NMR (125 MHz, DMSO-$d_6$): 174, 156.0, 155.9, 144, 141, 133, 128, 127.0, 126.9, 125.1, 125.0, 120.1, 119.9, 65, 64, 53.54, 53.50, 47, 28, 26. $R_t$ (10-100%): 21.9 min. *m/z* calcd for $C_{24}H_{26}N_2O_6$: 438.18; found 461.5 [M + $Na^+$].

**$N^{\alpha}$-Alloc-$N^{\varepsilon}$-Fmoc-L-lysine.** $N^{\alpha}$-Alloc-$N^{\varepsilon}$-Boc-L-lysine (0.54 g, 1.64 mmol) was subjected to Boc deprotection and subsequently reprotected with Fmoc-OSu (0.55 g, 1.64 mmol) and gave a white foam as sufficiently pure product (0.67 g, 92%). $^{1}$H NMR (250 MHz, DMSO-$d_6$): δ 12.53 (s, 1H), 7.90 (d, 2H), 7.69 (d, 2H), 7.38 (m, 6H), 5.89 (m, 1H), 5.24 (dd, 2H), 4.48 (d, 2H), 4.27 (m, 3H), 3.91 (m, 1H), 2.97 (br d, 2H), 1.6 (br m, 2H), 1.37 (br m, 4H). $^{13}$C NMR (63 MHz, DMSO-$d_6$): 174.4, 156.5, 156.2, 144.4, 141.2, 134.1, 128.1, 127.5, 125.6, 120.5, 117.4, 64.8, 60.2, 47.3, 28.4, 23.3, 14.5. $R_t$ (10-100%): 22.5 min. *m/z* calcd for $C_{25}H_{28}N_2O_6$: 452.19; found 475.5 [M + $Na^+$].

***N*-Fmoc-5-aminopentanoic acid.** 5-aminopentanoic acid (0.35 g, 3 mmol) was protected with *o*- Fmoc-OSu (1.01 g, 3 mmol) and gave a slightly yellow, sticky oil as sufficiently pure product (0.92 g, 90%). $^{1}$H NMR (500 MHz, DMSO-$d_6$): δ 12.02 (br s, 1H), 7.86 (d, 2 H), 7.67 (d, 2H), 7.39 (t, 2H), 7.31 (t, 2H), 7.26 (t, 1H), 4.28 (d, 2H), 4.19 (t, 1H), 2.97 (dd, 2H), 2.19 (t, 2H), 1.43 (br m, 4H). $^{13}$C NMR (125 MHz, DMSO-$d_6$): 174.9, 156.7, 144.4, 141.2, 128.0, 127.5, 125.6, 120.5, 65.6, 64, 47.2, 33.8, 29.3, 22.2. $R_t$ (10-100%): 20.73 min. *m/z* calcd for $C_{20}H_{21}NO_4$: 339.15; found 701.3 [2M + $Na^+$].

**Labeling of *cyclo*(-D-Tyr$^1$-Arg$^2$-Arg$^3$-Nal$^4$-Gly$^5$) with $^{125}$I**

The radioabeled competitor used in the binding assay, *cyclo*(-D-Tyr$^1$[$^{125}$I]-Arg$^2$-Arg$^3$-Nal$^4$-Gly$^5$) ($^{125}$I-CPCR4) was produced as described elsewhere. Briefly, Iodogen iodination reagent (“IODO-GEN Iodination Reagent”, Perbio Science Deutschland GmbH) was dissolved in dry dichloromethane, transferred into an Eppendorf tube and carefully dried with argon gas. To *cyclo*(-D-Tyr$^1$-Arg$^2$-Arg$^3$-Nal$^4$-Gly$^5$), dissolved in phosphate buffered saline (PBS) and transferred into an Iodogen coated cup, sodium $^{125}$I-iodide was added. After reaction at room temperature the product peptide was purified by radio-HPLC. For all binding assays, a pre-calculated volume of ($^{125}$I-CPCR4) stock solution was diluted with PBS to yield 2 mL solution with an activity concentration of 100,000 cpm 25 $\mu L^{-1}$.

**Receptor Binding Assays.** Competition binding experiments were performed using the Jukart cell line. In brief, cells were resuspended in PBS/0.2 % BSA. A total of 200 µL of the suspension containing 400,000 Jurkat cells were incubated with 25 µL of, $^{125}$I-CPCR4 solution, (containing 3.1 kBq, approx. 0.1 nM) and 25 µL of the tested peptides at concentrations of $10^{-11}$ to $10^{-5}$ M. Nonspecific binding was determined in the presence of 1 µM cyclo(-D-Tyr$^1$-Arg$^2$-Arg$^3$-Nal$^4$-Gly$^5$). After shaking for 2 h at room temperature, the incubation was terminated by centrifugation at 1300 rpm for 5 min. Cell pellets were washed twice with cold PBS. Cell bound radioactivity was determined by using a 1480 Wizard3 gamma-counter from Wallac (Turku, Finland)). Experiments were repeated 2-3 times in triplicates. $IC_{50}$ values of the compounds were calculated by nonlinear regression using GraphPad Prism (GraphPad Prism 4.0 Software, Inc., San Diego, CA, USA). Each data point is the average of three determinations.

**Labelling with $^{68}$Ga.** Due to its high affinity to CXCR4, $^{68}$Ga-**2c** was selected for an initial *in vivo* study. For this purpose, Gallium-68 ($e^+$ = 89%, $t_{1/2}$ = 68.1 min, $E_{ß+max}$ = 1.90 MeV) was eluted from a commercially available Ge-68/Ga-68 generator (iThemba, South Africa) by diluted HCl. The fraction with the highest activity, approx. 1.2 mL with > 80% of the entire eluted activity, was used for labeling of **2a** using a commercially available fully automated labeling module (Gallelut-Synthesizer, Scintomics GmbH, Fürstenfeldbruck, Germany). After the pH of the eluate was adjusted with a suitable amount of HEPES (4-(2-hydroxyethyl)-1-piperazineethanesulfonic acid) (930 µL, 600 mg HEPES 0.5 $mL^{-1}$ $H_2O$), 20 µg (15 nmol) of the peptide was added. After reaction for 5 min at 95 °C, quality control of the product was carried out by radio thin layer chromatography (TLC) on Silica gel 60-plates using 0.5 µL of product solution on 2 different TLC systems: a) eluent TLC-1: 0,1 M sodium citrate (5.882 g tri-sodium citrate dihydrate in 200 ml ultrapure water); eluent TLC-2: (1/1, v/v) methanol / 1M ammonium acetate (15.461 g ammonium acetate in 200 mL ultra pure water). Using the TLC method 1, the product and Ga-colloid stay at the starting point, whereas free $Ga^{3+}$ moves with front. Using the TLC method 2, uncomplexed Ga-species stay at starting point, whereas the labeled peptide moves with front. The quality control of the labelled peptide was also achieved by RP-HPLC. HPLC analysis was performed on a Semi RP18 Multosphere Column (250 x 100 mm) applying a linear gradient system at a 5 mL/min flow rate from 49% B to 60% B in 20 min, where solvent A = 0.2 M ammonium formate and solvent B = MeOH. The detection of the peptides was performed via UV absorption at 220 nm.
Standard reaction conditions (15 nmol of peptide, starting activities of 1.1-1.3 GBq) yielded $^{68}$Ga-CPCR4-2 in 50% radiochemical yield (based on initial $^{68}$Ga activity, d.c. (decay corrected)) and >95% radiochemical purity (as confirmed by HPLC/TLC) within 17 min.

All animal experiments were approved by local authorities and are in compliance with the institutions guidelines.

**Biosdistribution Studies.** Athymic nude mice (average weight 30 g) were obtained from Charles River, Germany. The animals were inoculated subcutaneously into the left shoulder with OH1 h-SCLC cells ($5x10^6$ cells/animal). Tumors were allowed to grow for three weeks. Tissue distribution studies of the $^{68}$Ga labeled tracer were carried out after the intravenous administration of 0.1 mL (60 - 80 µCi, 0.7-0.8 µg of total peptide) of the labeled product via the tail vein. Animals were sacrificed at 1 h p.i.. Subsequently, the tissues and organs were weighted, the radioactivity was counted in a 1480 Wizard3 gamma-counter from Wallac (Turku, Finland), and the % ID/g of each organ or tissue was calculated. Specificity of binding was demonstrated by co-injection of unlabeled *cyclo*(-D-$Tyr^1$-$Arg^2$-$Arg^3$-$Nal^4$-$Gly^5$) (50 µg/mouse; n=4).

**PET-Camera Imaging.** Mice (~ 30 g) were anaesthetized using isoflurane anesthesia and injected with 230 - 300 µCi (6 - 8 MBq, 2-3 µg of total peptide) of [$^{68}$Ga]2c via the tail vein in a volume of 300 µL of PBS. For the blocking experiments, co-injection of the tracer with 50 µg of the unlabeled *cyclo*(-D-Tyr$^{1}$-Arg$^{2}$-Arg$^{3}$-Nal$^{4}$-Gly$^{5}$) took place. PET scans were performed using an Inveon Siemens PET Scanner. 90 min after the injections the mice were placed on a platform inside of the microPET scanner and static images were acquired for 20 min. The images were reconstructed by a two-dimensional ordered subsets expectation maximum (2D-OSEM) algorithm, and no correction was applied for attenuation. Images analysis was done using Inveon software and the images are presented in Figure 3 **A** (unblocked) and **B** (blocked). The results were calculated as Bq/mL.


[1] S. Mizukami, R. Takikawa, F. Sugihara, Y. Hori, H. Tochio, M. Walchli, M. Shirakawa, K. Kikuchi, *Journal of the American Chemical Society* **2008**, *130*, 794.
[2] J. V. Aldrich, V. Kumar, United States of America, **2003**, p. 14.
[3] R. Haubner, B. Kuhnast, C. Mang, W. A. Weber, H. Kessler, H. J. Wester, M. Schwaiger, *Bioconjugate Chemistry* **2004**, *15*, 61.
[4] O. Demmer, I. Dijkgraaf, M. Schottelius, H. J. Wester, H. Kessler, *Organic Letters* **2008**, *10*, 2015.
[5] H. Kessler, B. Kutscher, *Liebigs Annalen der Chemie* **1986**, 914.
[6] C. Gilon, C. Mang, E. Lohof, A. Friedler, H. Kessler, in *Houben-Weyl: Methods of Organic Chemistry, Vol. E 22b* (Eds.: M. Goodman, L. Felix, L. Moroder, C. Toniolo), Thieme Verlag, Stuttgart, **2003**, pp. 461.
[7] O. Demmer, A. O. Frank, H. Kessler, in *Design of Peptides and Proteins for Biopharmaceutical Applications : Applications for Therapeutic Agents and Biomedical Research* (Ed.: K. J. Jensen), Wiley, **2009**, pp. 133.
